# Leadership in public administration amid crisis: a meta-analysis of leadership styles and crisis management related outcomes

**DOI:** 10.3389/fpsyg.2026.1803778

**Published:** 2026-05-18

**Authors:** Chan Nyein Aung, Xinwen Zhang

**Affiliations:** College of Public Administration, Nanjing Agricultural University, Nanjing, China

**Keywords:** crisis management, leadership style, meta-analysis, public administration, transformational leadership

## Abstract

Strong leadership is essential for efficient crisis management within public administration throughout global poly crises to alleviate the crisis's effect on individuals and bolster organizational resilience. This meta-analysis examined the correlations between leadership styles and crisis management outcomes in the context of public administration. Following the PRISMA guidelines, the study conducted a systematic search of the eight databases for studies published till December 2025. Methodological quality was evaluated using the Joanna Briggs Institute (JBI) critical appraisal tools. The final analysis included 50 studies, resulting in 72 effect sizes, 26 leadership styles, and representing 21815 participants. A random-effects model was employed to conduct the main meta-analysis, moderator analyses, and publication bias tests. The findings discovered that transformational leadership is the prevalent style in public administration amid crisis management. The meta-analysis revealed that leadership styles exhibited a significant positive correlation with crisis management outcomes, including crisis phases, sense-making, meaning-making, and learning-related outcomes. The moderating effects of the administrative subfields, administrative continent, types of crises, and hierarchy level significantly influence the nexus between leadership styles and crisis management outcomes. No substantial publication bias was detected (Egger's test, *p* = 0.29). This meta-analysis highlights the significance of leadership in crisis management within public administration and shows the necessity for context-specific leadership strategies adapted to the crisis environment and institutional framework.

**Systematic review registration:** https://osf.io/qt98p/overview, Open Science Framework (OSF).

## 1 Introduction

Nowadays, with a wide range of rapidly worsening crises such as pandemics, natural disasters, civil conflicts, and technology failures, the role of public administration (PA) leadership is more important than ever before (Asamoah and Bigodza, 2025; Miao et al., 2025). Amidst these turbulent situations, public officials are positioned at the front lines of public administration, maintaining institutional continuity and public trust, confronting significant challenges in managing crises. Leadership is universally acknowledged as an essential and effective strategy for addressing the challenges and opportunities that emerge in the global context (Chiwisa, 2024). Existing research consistently suggests that leadership has a direct impact on crisis management effectiveness (Hasan and Rjoub, 2017) and that the right leadership style is crucial in managing various types of crises in public administration (Asamoah and Bigodza, 2025).

Crisis management is pivotal in public administration because different types of crises, such as pandemics, economic, and natural disasters, can disrupt governance systems, weaken organizational resilience, disrupt stability, facilitate policy changes, and generate administrative, financial, and operational problems (Guy and Jon, 2010; Miao et al., 2025; Olbassali et al., 2022). More broadly, crises affect about 300 million people worldwide each year and place extraordinary demands on public institutions (Crises, n.d.). Crisis management is a continuous process aimed at preventing or mitigating negative impacts through activities conducted before, during, and after a crisis (Hede, 2018). Therefore, for public administration leaders, this means that effective crisis management depends not only on technical capacity or procedural compliance but also on leadership approaches that can mobilize people, interpret uncertainty, coordinate action, and support adaptation over time.

Scholars have extensively attempted to identify effective leadership styles in crisis management. The successful execution of crisis management is shaped by diverse leadership styles, such as transformational, transactional, adaptive, and authoritarian (Hazaa et al., 2021; Miao et al., 2025). Transformational leadership is often associated with inspiration, trust-building, and the articulation of collective purpose, all of which may be especially valuable under crisis conditions (Chiwisa, 2024). Transactional leadership focuses on organizational structure and compliance with established protocols, ensuring stability amid crises (Chiwisa, 2024). Crisis leadership is the process of leading at times of crisis, such as prior to a crisis, during a crisis, and after the worst effects of a crisis (Wu et al., 2021). Crisis leadership focuses on how leaders influence various stakeholders during crises and how they respond to various types of crises (Collins et al., 2022). Adaptive leadership exhibits flexibility, agility, and responsiveness to unexpected difficulties, thereby promoting organizational learning, facilitating adaptation, and permitting improvisation in promptly changing environments (Ansell et al., 2022; Miao et al., 2025). The authoritarian leadership possesses exclusive decision-making authority and completely supervises and governs the followers. The autocratic leader offered clear direction and facilitated rapid decision-making both during and after the crisis (Labib, 2024). These leadership styles possess unique characteristics that facilitate the effective execution of key crisis management processes such as sense-making, decision-making, meaning-making, and learning.

Researchers have examined the influence of leadership styles on crisis management across various dimensions; (i) crisis phases, specifically three phases (Tokakis et al., 2018), four stages (Frank and Lucky, 2025), and five stages (Alkhawlani et al., 2019; Maghdid et al., 2022); and (ii) executive responsibilities in crisis management, particularly, trust (Mao et al., 2025), communication (AlAjmi, 2022), resilience enhancement (Aravidou et al., 2025), psychological safety (Ma et al., 2021; Zhao et al., 2020), knowledge sharing (Rafique et al., 2022; Mao et al., 2025), and agility (Khoshlahn and Ardabili, 2016). However, these isolated research findings have led to confusion and fragmentation among academics and practitioners in selecting the most effective leadership approach in crisis contexts. The research yields significant insights; nonetheless, it has yet to furnish a comprehensive and cohesive explanation of the most effective leadership styles for crisis management in public administration, the conditions in which they operate, and the associated outcomes.

A meta-analysis can provide a comprehensive insight into the relationship between various leadership styles and crisis management-related outcomes. Importantly, leadership effectiveness in crisis contexts is unlikely to be universal. The requirements will differ based on factors including the crisis's type, cultural and regional context, the organization's type, the organizational hierarchy, and the event's scope (Chiwisa, 2024). Public administration encompasses diverse subfields with distinct goals, structures, and operational demands, and leadership practices may therefore differ across administrative domains (Backhaus and Vogel, 2022). Similarly, administrative traditions and cultural context significantly shape leadership expectations and crisis responses (House et al., 2004; Backhaus and Vogel, 2022). Crisis type also matters, as different crises may require different leadership responses (Wu et al., 2021); for example, the financial crisis was accompanied by a notable rise in directive leadership (Stoker et al., 2018); strategic leadership is crucial during organizational crises (Schaedler et al., 2021). Additionally, leadership effect may vary across hierarchical levels because senior and junior public officials often perform different roles and bear different responsibilities in crises (Ansari and Naeem, 2010).

The current study examines the relationship between leadership styles and crisis management-related outcomes in public administration, including sense-making, decision-making, meaning-making, and learning. These outcomes are treated as theoretically meaningful and interrelated dimensions of crisis management because they reflect how public organizations interpret crises, make decisions under uncertainty, communicate meaning to stakeholders, and adapt through reflection and learning. The specific objectives are as follows: (i) to evaluate the leadership styles utilized in public administration amid crisis management, (ii) to systematically compile empirical evidence regarding the correlation between leadership styles and crisis management-related outcomes, and (iii) to analyze moderators that influence the relationship between leadership styles and crisis management outcomes.

This meta-analysis contributes to the literature in three ways. First, it provides a comprehensive empirical synthesis of the relationship between leadership styles and crisis management-related outcomes in public administration. Second, it offers a conceptual framework by organizing crisis management outcomes into theoretically meaningful categories linked to core crisis management processes. Third, it promotes the contingent nature of leadership effectiveness by examining key moderators, thereby offering a more context-sensitive understanding of leadership in crises. This study contributes to leadership theory and addresses the practical question of which leadership strategies are most effective for public administration amid a crisis.

## 2 Literature review and hypotheses

### 2.1 Leadership in public administration

Leadership in public administration has evolved alongside broader developments in leadership theory. Van Wart (2003) indicated that leadership theory commenced with the great man theory, trait theory (1900-1948), the contingency model (1948-1980), the transformational leadership theory (1978-present), servant leadership theory (1977-present), and the multifactorial theory (1990-present) (Van Wart, 2003). Over the past 10 years, the concept of humble leadership has gained attention among leadership scholars (Liden et al., 2024). During the era of advanced information technology, digital leadership was embraced by many scholars (Avolio et al., 2000).

Across this broad literature, leadership may be examined through multiple lenses, including trait theories, behavioral theories, contingency theories, the relational approach (i.e., leader-member exchange), and leadership styles (Liden et al., 2024). Among these, leadership styles have become particularly prominent in public administration research because they capture relatively stable patterns in how leaders influence, guide, and interact with followers in organizational settings (Liden et al., 2024). Leadership styles are defined as the constant combinations of characteristics and behaviors exhibited by leaders (Backhaus and Vogel, 2022). Public-sector scholarship has therefore increasingly examined how leadership styles affect employee attitudes, organizational processes, and administrative performance (Araya-Orellana, 2021).

This question becomes especially important in crisis contexts. Recent global crises, including pandemics, natural disasters, terrorism, and technological disruptions, have highlighted the need for leadership approaches that are not only effective under routine conditions but also appropriate under uncertainty, urgency, and public pressure (Suharyanto and Lestari, 2020). In this context, leadership style denotes the various methods utilized by leaders to inspire, direct, and supervise subordinates in pursuit of organizational goals (Abodunde et al., 2017). However, contingency theories propose that no single leadership style is universally effective across all situations, and the selection of a leadership approach is contingent upon different factors, including the abilities and situations of the followers, task demands, and environmental conditions (Chiwisa, 2024).

Public administration research has accordingly examined a wide range of leadership styles, including transformational, transactional, authentic, humble, destructive, entrepreneurial, ethical, servant, shared, paradoxical, digital, crisis, adaptive leadership, etc. (Backhaus and Vogel, 2022; Adie et al., 2024; Mao et al., 2024; Su, 2024; Liden et al., 2024). However, in a crisis management context, it is unclear whether all leadership styles work as well. This uncertainty is particularly salient in public administration, where leaders must operate within formal rules, institutional constraints, multiple stakeholders, and heightened accountability. A clearer synthesis is therefore needed to determine which leadership styles are most strongly associated with crisis management-related outcomes in public administration.

### 2.2 Crisis management in public administration

Crisis management plays a crucial role in public administration because governments and public institutions are expected to prevent, prepare for, respond to, and recover from crises that threaten both organizational functioning and public welfare. Crisis management is defined as the procedures, strategies, and practices utilized by public administration institutions to prevent, prepare for, respond to, and recover from emergencies and crises (Chiwisa, 2024; Miao et al., 2025). Additionally, “Crisis management is the process by which a government agency works to avoid crises or events that may be harmful to the organization or to the public at large and works to mitigate the effects of any crises that may occur” (Turpin, 2016). These definitions underscore that crisis management is not limited to emergency response alone, but encompasses a broader governance process extending across pre-crisis, crisis, and post-crisis periods.

The crisis management literature in public administration has expanded in response to increasing global instability and the growing complexity of crisis environments. Public administration authorities employed different crisis management strategies depending on institutional context, available resources, and the nature of the crisis itself (Korolchuk et al., 2020). However, crisis management research can be identified into the crisis phases and executive tasks. Crisis researchers have developed types of crises into various techniques contingent upon distinct situations. According to crisis intervention, the types of crises include situational, maturational or developmental, and adventitious or social crises (Danna and Bennett, 2011). Hutchins (2008) attempted to categorize crises into two distinct types: “natural crises (hurricanes, earthquakes, and fire)” and “human-induced crises (bribery, corruption, scandal, and terrorist attack)”. United Nations (UN) standards recognize both natural disasters and man-made crises (Kapucu and Ustun, 2017). In the public administration context, more recent studies commonly refer to broad categories such as pandemics (COVID-19, SARS, Zika, etc.), natural crises (floods, earthquakes, tsunamis, etc.), civil conflict (terrorism, wars, crime, etc.), and technological failures (Hartmann, 2012; Alzoubi and Jaaffar, 2020; Miao et al., 2025). These distinctions matter because different types of crises may create different operational demands and may therefore require different leadership responses.

Researchers have developed several crisis management models to effectively navigate the different stages of a crisis, including Smith's (1990) three-stage model, which highlights pre-crisis, crisis, and post-crisis; Myers' (1993) four-stage model, which underscores normal operations, emergency response, interim processing, and restoration; Pearson and Mitroff (1993) five-stage model, which emphasizes signal detection, preparation and prevention, damage containment, recovery, and learning (Crandall et al., 2020); organizational culture in crisis management model (Veil, 2010); and the Situational Crisis Communication Theory (SCCT), which concentrates on strategic communication during crises (Coombs, 2022). All models were attempted to identify various phases of crisis management, offering a more detailed, step-by-step analysis of the process.

Beyond crisis phases, scholars have also identified a set of executive tasks that are central to crisis management. The executive tasks can be identified into five elements, such as sense making, decision making, meaning making, terminating, and learning (Boin et al., 2016). Among these, sense-making, decision-making, meaning-making, and learning are especially relevant to the present study because they capture core processes through which public organizations interpret crises, formulate and coordinate responses, communicate direction and legitimacy, and adapt after disruption. These characteristics are conceptually interconnected rather than independent, collectively reflecting fundamental elements of crisis management performance in public administration. These tasks are especially useful because they connect leadership directly to how crises are interpreted, managed, communicated, and reflected upon (Boin et al., 2013). In practice, crisis management is also shaped by broader factors such as communication and social media, leadership, knowledge, governance, information technology, strategic planning, and professional entities (Hazaa et al., 2021). Nevertheless, leadership remains one of the most consistently emphasized drivers of effective crisis management. Effective leadership can help address uncertainty, mobilize coordination, and sustain organizational functioning, whereas ineffective leadership may worsen crisis consequences or delay response (Chiwisa, 2024). Consequently, the relationship between leadership styles and crisis management-related outcomes needs to be examined to attain in-depth knowledge.

### 2.3 Relationship between leadership styles and crisis management

Leadership styles are a pivotal determinant of the success or failure of crisis management effectiveness in public administration (Chiwisa, 2024; Hazaa et al., 2021). Effective leadership addresses problems like uncertainty, resource constraints, communication failures, and the necessity for rapid decision-making. It promotes adaptation, efficient communication, and collaboration (Labib, 2024). In times of crisis, public leaders need to employ effective and suitable leadership styles to effectively navigate crisis phases and to enhance sense-making, decision-making, meaning-making, and learning while implementing crisis management tasks (Boin et al., 2016). In this sense, leadership is relevant not only to overall crisis management performance but also to specific crisis management-related outcomes.

The relationship between leadership styles and crisis management outcomes can be better explained through a multi-theoretical perspective. First, contingency theory suggests that leadership effectiveness depends on situational fit, meaning that different leadership styles may be more effective under different crisis conditions, administrative contexts, and organizational roles (Fiedler, 1964). Second, sensemaking theory explains how leaders help organizations interpret ambiguous events, reduce uncertainty, and establish shared understandings that guide coordinated action (Maitlis and Christianson, 2014). Third, social exchange theory suggests that leadership styles characterized by trust, support, and fairness strengthen follower commitment, cooperation, and psychological safety, which are especially relevant to the meaning-making and learning process (Cropanzano and Mitchell, 2005). Fourth, situational crisis communication theory highlights the role of leadership communication in shaping stakeholder understanding, legitimacy, and crisis response effectiveness (Coombs, 2022).

Moreover, the Leader–Member Exchange (LMX) theory suggests that leaders employ distinct management styles for each subordinate. In turn, each relationship and associated management style generates distinct responses and attitudes in subordinates, influencing various performance behaviors (Fein and Tziner, 2021). During crises such as COVID-19, high-quality relationships between supervisors and followers can reduce the likelihood of employee turnover, although organizational support may be less important during these periods (Garcia-Pereyra et al., 2025). Therefore, high-quality relationships, characterized by trust, mutual support, and effective communication, can strengthen psychological safety, coordination, and shared understanding, ultimately improving crisis management-related outcomes. Taken together, these theories suggest that sense-making, decision-making, meaning-making, and learning are not isolated constructs, but interrelated dimensions of crisis management. They reflect how organizations interpret crises, make critical choices, coordinate responses, sustain legitimacy, and adapt over time. Accordingly, this study treats these four dimensions, together with broader crisis-phase and overall crisis management indicators where reported in the primary studies, as key crisis management-related outcomes through which the effectiveness of leadership styles can be assessed.

More specifically, these dimensions represent interrelated crisis-management processes through which leadership influences organizational outcomes. Sense-making pertains to the interpretation of ambiguous and dynamic events; decision-making entails the formulation and coordination of prompt responses; meaning-making involves conveying direction and legitimacy to pertinent stakeholders; and learning signifies the translation of crisis experiences into future adaptation and readiness. Consequently, these dimensions are categorized as crisis management outcomes, as they encompass complementary mechanisms by which leadership influences crisis response and recovery in public organizations (Maitlis and Christianson, 2014; Boin et al., 2016; Jong, 2017).

To enhance conceptual clarity, leadership styles must be differentiated by their foundational assumptions and behavioral mechanisms, as they are unlikely to exert uniform influence across conditions of crisis. Transformational leadership may be especially relevant in uncertain and evolving crises because it supports vision, trust, adaptation, and learning, whereas more directive approaches may be more useful when crises require rapid coordination and centralized action. Relational forms of leadership may be particularly valuable when crisis response depends on legitimacy, inclusion, and sustained cooperation. These distinctions reinforce a contingency-based view in which leadership effectiveness depends on the fit between leadership behavior and the specific demands of the crisis and administrative setting (Table 1).

**Table 1:** Leadership styles and their theoretical relevance to crisis management-related outcomes.

Leadership style	Definition	Crisis context	Crisis outcome	Crisis management contribution	Key references
Transformational leadership	Inspires followers through vision, intellectual stimulation, and motivation to exceed expectations	Complex, uncertain, and prolonged crises	Sense-making; learning; overall crisis management	Helps interpret uncertainty, build shared direction, and promote adaptation and learning	Bass and Avolio, 2004; Bhaduri, 2019; Purnomo et al., 2021; Rafique et al., 2022
Transactional leadership	Emphasizes task structure, rewards, and performance monitoring	Structured crisis settings require control and compliance	Decision-making; crisis phases	Supports coordination, procedural clarity, and implementation efficiency	Bass and Avolio, 2004; Bowers et al., 2017
Authoritarian leadership	Relies on centralized authority and directive decision-making	High-risk, urgent, and time-sensitive crises	Decision-making; crisis phases	Enables rapid decisions, clear command, and strict resource control	Labib, 2024
Democratic leadership	Encourages participation and shared decision-making	Collaborative crisis settings involving multiple stakeholders	Decision-making; meaning-making	Promotes inclusiveness, shared responsibility, and stakeholder coordination	Labib, 2024
Crisis leadership	Leadership adapted to crisis conditions, emphasizing rapid action, clear communication, and effective resource use	Acute crises, such as disasters and pandemics	Sense-making; decision-making; meaning-making; learning; overall crisis management	Supports crisis interpretation, coordinated action, resilience, and recovery	Jing and Yang, 2025; Labib, 2024
Authentic leadership	Emphasizes trust, empathy, transparency, ethics, and self-awareness	Crises requiring legitimacy, trust, and reassurance	Meaning-making	Builds trust, credibility, morale, and psychological safety	Atilgan et al., 2025
Servant leadership	Prioritizes followers' needs, development, and wellbeing	Social crises and human-centered service settings	Meaning-making; learning	Strengthens wellbeing, collaboration, and resilience while supporting adaptation	Yikilmaz et al., 2024
Digital leadership	Uses digital tools and technologies to guide and coordinate organizations	Technology-driven crises, including pandemics	Decision-making; sense-making; learning	Supports data-driven decisions, digital coordination, and knowledge sharing	Zhao et al., 2025
Inclusive leadership	Encourages openness, diversity, and participation	Crises affecting diverse or vulnerable populations	Sense-making; meaning-making	Improves psychological safety, engagement, and shared understanding	Zhao et al., 2020
Responsible leadership	Emphasizes ethics, stakeholder engagement, and accountability	Crises with strong social and ethical implications	Meaning-making	Strengthens legitimacy, accountability, and stakeholder trust	Obuobisa-Darko et al., 2023

#### 2.3.1 Leadership styles and crisis management related outcomes

Several studies suggest that leadership is positively associated with crisis management performance at an overall level. For example, crisis leadership (Alharthi and Khalifa, 2019), responsible leadership (Obuobisa-Darko et al., 2023), and distributed leadership (Alene, 2022) have all been linked to stronger crisis management capacity or performance in public-sector or crisis-related settings. More broadly, resilience-oriented perspectives suggest that leadership can enhance organizational capacity to withstand and adapt to disruption by fostering relational coordination, collective interpretation, and supportive communication (Teo et al., 2017). These findings suggest that leadership style should, on average, be positively associated with crisis management-related outcomes. Therefore, we formulated the following hypotheses:

*Hypothesis 1 (H1): The leadership style is positively correlated with overall crisis management outcomes*.

#### 2.3.2 Leadership styles and crisis phases related outcomes

Pearson and Mitroff (1993) believed that a comprehensive understanding of crisis and its phases is a more suitable approach to effective crisis management. Crisis management related to crisis phases is improved by pre-crisis, during-crisis, and post-crisis strategies, as well as signal detection, preparation and prevention, damage containment, recovery, and learning strategies (Pearson and Mitroff, 1993). Empirical evidence showed that leadership styles influence the phase of crisis management by influencing preparedness, coordination, response implementation, and post-crisis adjustment. Transformational, authoritarian, and democratic leadership significantly enhance crisis management, encompassing the prevention and planning phase, implementation phase, evaluation phase, and feedback phase (Gharib and Elnahas, 2021). Similarly, distributed leadership has been linked to positive crisis management across pre-crisis, during crisis, and post-crisis (Alene, 2022). And authentic leadership is strongly positively correlated with crisis-related outcomes (Atilgan et al., 2025). Therefore, these findings support the expectation that leadership styles will be positively related to crisis-phase-related outcomes.

*Hypothesis 2 (H2): The leadership style is positively correlated with crisis-phase-related outcomes*.

#### 2.3.3 Leadership styles and sense-making related outcomes

Sense-making refers to the process through which individuals and organizations interpret ambiguous events and construct plausible understandings that guide action (Daft and Weick, 1984). In crisis contexts, sense-making is essential because leaders must help reduce ambiguity, coordinate interpretation, and support collective understanding under uncertainty (Boin et al., 2016; Baran and Scott, 2010). Outcomes related to sense-making may include psychological safety, resilience, reduced ambiguity, shared understanding, and related interpretive capacities. Prior studies suggest that crisis leadership substantially enhances organizational sense-making and organizational resilience (Jing and Yang, 2025). Inclusive leadership is positively correlated with psychological safety and inversely associated with the psychological distress of healthcare personnel during the pandemic (Zhao et al., 2020). Digital leadership enhances community resilience during crises by offering digital knowledge and technology infrastructure (Zhao et al., 2025). A substantial negative association existed between servant leadership and emotional exhaustion during natural disasters and climate change (Yikilmaz et al., 2024). Thus, the following hypothesis was established:

*Hypothesis 3 (H3): The leadership style is positively correlated with sense-making-related outcomes*.

#### 2.3.4 Leadership styles and decision-making related outcomes

Decision-making is a core task of crisis management because crises often require rapid judgment, prioritization, coordination, and action under time pressure and uncertainty (Argyris and Schön, 1978; Boin et al., 2016). Leadership may influence decision-making by shaping how information is processed, how alternatives are evaluated, and how collective action is coordinated. Prior research suggests that transformational leadership can foster strategic understanding, resilience, and adaptive decision processes in prolonged crises (Purnomo et al., 2021). Additionally, authoritarian and collaborative leadership can improve confrontation and organizing, whereas transformational leadership can facilitate compromise and organizing in conflict management (Aravidou et al., 2025). So, the following hypothesis was formulated:

*Hypothesis 4 (H4): The leadership style is positively correlated with decision-making-related outcomes*.

#### 2.3.5 Leadership styles and meaning-making related outcomes

Meaning-making refers to the process through which leaders and organizations construct, communicate, and sustain shared interpretations that preserve legitimacy, trust, morale, and collective orientation during crisis (Varney, 2009). In public administration, meaning-making is especially important because crisis responses are often judged not only by operational effectiveness but also by public communication, credibility, and perceived legitimacy. Outcomes related to meaning-making may therefore include trust, reputation, wellbeing, satisfaction, credibility, and related indicators. Empirical studies have shown that charismatic leadership communication correlates positively with perceived organizational reputation (Jamal and Bakar, 2015). In addition, crisis leadership positively influences trust (Mao et al., 2025), and self-sacrificial leadership enhances workplace thriving and wellbeing of employees amid the pandemic (Huang and Zhou, 2023). Several leadership styles, including authoritarian, democratic, transformational, and transactional leadership, exhibit a substantial positive correlation with quality of work life (Ebrahim et al., 2022). Therefore, these considerations result in the formulation of the following hypothesis:

*Hypothesis 5 (H5): The leadership style is positively correlated with meaning-making related outcomes*.

#### 2.3.6 Leadership styles and learning-related outcomes

Learning is a critical component of crisis management because effective crisis response requires reflection, adaptation, improvisation, and the capacity to improve future performance (Comfort, 1999). Learning-related outcomes may include innovation, knowledge sharing, team cohesion, performance improvement, workplace buoyancy, and related adaptive capacities. Prior research has found that transformational leadership is positively correlated with knowledge sharing and innovative work behavior amid the pandemic (Rafique et al., 2022). Sharing knowledge and public service motivation serve as beneficial mediators between crisis leadership and civil servant performance during the pandemic response (Mao et al., 2025). Servant leadership enhances the workplace buoyancy among disaster response workers (Yikilmaz et al., 2024). Thus, we formulated the following hypothesis:

*Hypothesis 6 (H6): The leadership style is positively correlated with learning-related outcomes*.

### 2.4 Moderators analyses

This study suggests that the effectiveness of leadership in crisis management is contingent upon context rather than being universally applicable. Moderating factors play a vital role in leadership effectiveness and crisis management-related outcomes studies. Previous meta-analytical research in public administration and leadership research has employed administrative subfields, administrative traditions, data sources, and research design (Backhaus and Vogel, 2022), cultural value, organizational types, and measurement scales (Bao et al., 2024). Building on this logic, the present study focuses on four contextual moderators that are especially relevant to public administration crisis research: administrative subfields, administrative continent, crisis type, and hierarchy level.

#### 2.4.1 Administrative subfields

Administrative subfields are important because public-sector domains differ in organizational goals, professional norms, task structures, and levels of formalization. Public health, public education, general administration, law enforcement/military, and general public service operate under different institutional demands, which may shape both leadership behavior and its consequences (Backhaus and Vogel, 2022). The theory of substitutes for leadership suggests that situational factors can improve, neutralize, or completely substitute for leadership (Kerr and Jermier, 1978). A previous study revealed that the strength of the relationship between leadership and outcomes differs only slightly across various administrative subfields. Specifically, the subfields of law enforcement/military and education have marginally weaker correlations with outcome variables compared to core administrative services (Backhaus and Vogel, 2022). Additionally, the public organization exhibits more robust correlations between transformational leadership and its outcomes than a semi-public organization (Bao et al., 2024). Researchers should consider the context of administrative subfields when examining leadership (Slyke and Alexander, 2006). Therefore, this study employed administrative subfields as a moderating variable in the leadership-outcomes relationship. In response to this justification, we propose H7.

*Hypothesis 7 (H7): Administrative subfields influence the relationship between leadership styles and crisis management outcomes*.

#### 2.4.2 Administrative continent

Leadership and its outcomes are also shaped by broader cultural and institutional environments. A prior analysis indicated that the correlation between leadership and its consequences varies across countries (Li et al., 2020). House et al. (2004) propose that leadership is based upon cultural factors. The country may affect the relationship of interest, although these connections may be complex. Additionally, a previous study revealed that the country only moderates the relationship between humble leadership–LMX linkage, demonstrating that the correlation is smaller in eastern countries compared to western countries (Luo et al., 2022). Crisis communication research likewise suggests that strategies that are effective in one cultural setting may not be equally effective in another (Wang and Laufer, 2019). Because public administration systems are embedded in broader regional traditions and governance contexts, it is reasonable to expect that the relationship between leadership styles and crisis management-related outcomes may vary across continents.

*Hypothesis 8 (H8): Administrative continent moderates the correlation between leadership styles and crisis management outcomes*.

#### 2.4.3 Crisis type

The type of crisis is another theoretically important moderator because different crises generate different demands, risks, and decision environments. The need for leadership during a crisis or extreme situation differs from that of normal times (Suharyanto and Lestari, 2020). Contingency leadership theory suggests that leadership effectiveness depends on the fit between leadership style and situational demands (Fiedler, 1964). The theory suggests that the effectiveness of leadership is contingent upon the specific environmental circumstances that develop in relation to a given action or behavior. Numerous crises, including natural catastrophes, terrorism, and the COVID-19 pandemic, necessitate outstanding leadership capable of adapting to prevailing conditions and circumstances (Suharyanto and Lestari, 2020). The various settings of crisis are essential in influencing and assessing governmental responses (Hood, 2021). Leadership styles and their outcomes can vary depending on the type of crisis. So, this analysis performs moderator analyses on types of crisis variables. In light of this rationale, we suggest H9.

*Hypothesis 9 (H9): The type of crisis affects the correlation between leadership styles and crisis management outcomes*.

#### 2.4.4 Hierarchy levels

Hierarchy level may also shape leadership effects in public administration because leaders at different organizational levels perform different functions and face different responsibilities. Senior leaders are often more involved in strategic direction, external coordination, and high-level crisis decisions, whereas junior or middle-level managers may focus more on supervision, implementation, and operational coordination. Many scholars have researched manager leadership styles across hierarchy levels inside an organization (Aras and Jufri, 2022). There exist notable disparities in leadership styles between senior and junior-level managers (Ansari and Naeem, 2010). Previous research has found differences in leadership style across hierarchy levels, with some evidence suggesting that senior managers are more likely to employ transformational or strategic leadership approaches, while lower-level managers may rely more on transactional or directive styles (Araya-Orellana, 2021; Ansari and Naeem, 2010). Therefore, these differences imply that hierarchy level may influence the observed relationship between leadership styles and crisis management-related outcomes.

*Hypothesis 10 (H10): Hierarchy levels influence the relationship between leadership styles and crisis management outcomes*.

### 2.5 Theoretical framework

**Figure 1:**
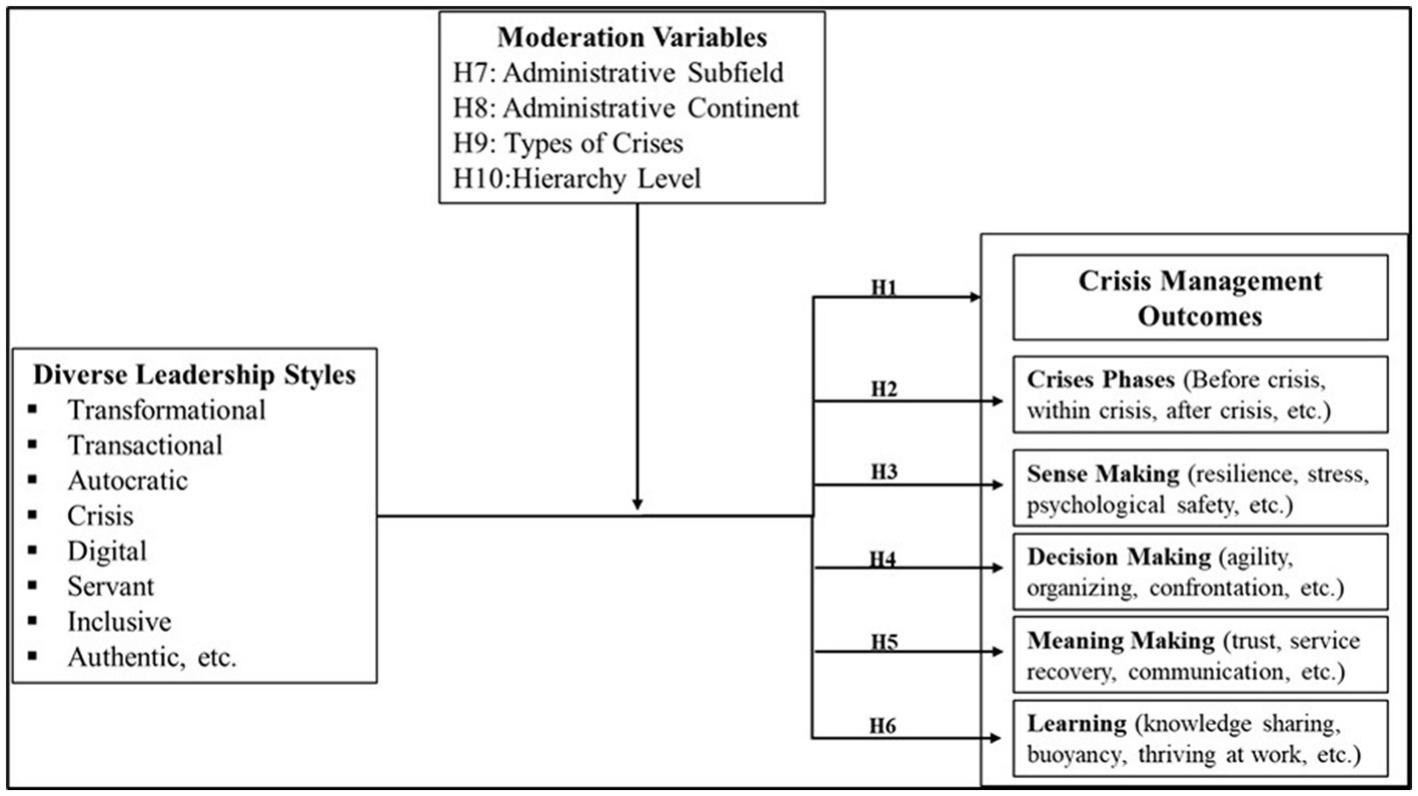
Meta-analytical theoretical framework.

Based on the foregoing literature, this study proposes a meta-analytic framework in which leadership styles are related to crisis management-related outcomes in public administration, including overall crisis management, crisis-phase-related outcomes, sense-making, decision-making, meaning-making, and learning. The framework further proposes that these relationships may vary depending on administrative subfields, administrative continent, crisis type, and hierarchy level. Figure 1 illustrates the meta-analysis theoretical framework.

## 3 Research method

### 3.1 Literature search and eligibility criteria

A protocol for this meta-analysis was preregistered on the Open Science Framework (OSF registration: https://osf.io/qt98p/). Following PRISMA 2020 guideline and recent meta-analyses (Backhaus and Vogel, 2022; Hazaa et al., 2021; Miao et al., 2025), we searched for published articles related to leadership style and crisis management in public administration across eight databases: Google Scholar, Sage, Science Direct, Scopus, Springer, Taylor & Francis, Web of Science, and Wiley. Consistent with previous investigation (Bao et al., 2024; Backhaus and Vogel, 2022; Miao et al., 2025; Lin et al., 2025), the search strategy combined the following terms: (“leadership” OR “leadership styles”) AND (“crisis” OR “crisis management”) AND (“public sector” OR “public administration”). The search covered studies available up to December 2025. The search strings for each database utilized in this study are presented in Table 2. PRISMA 2020 encourages for comprehensive reporting of the information sources utilized, the search strategy, and the study selection process, which guided the presentation of this section (Page et al., 2021).

**Table 2:** Database search strings.

Databases	Keywords used
Scopus	TITLE-ABS-KEY ((“leadership” OR “leadership styles”) AND (“crisis” OR “crisis management”) AND (“public sector” OR “public administration”))
Web of Science	TITLE-ABS-KEY ((“leadership” OR “leadership styles”) AND (“crisis” OR “crisis management”) AND (“public sector” OR “public administration”))
Science Direct	ALL ((“leadership” OR “leadership styles”) AND (“crisis management”) AND (“public administration”))
Springer	ALL ((“leadership” OR “leadership styles”) AND (“crisis management”) AND (“public administration”))
Sage	ALL ((“leadership” OR “leadership styles”) AND (“crisis management”) AND (“public administration”))
Taylor & Francis	ALL ((“leadership” OR “leadership styles”) AND (“crisis management”) AND (“public administration”))
Wiley	ALL ((“leadership” OR “leadership styles”) AND (“crisis management”) AND (“public administration”))
Google Scholar	TITLE ((“leadership” OR “leadership styles”) AND (“crisis” OR “crisis management”) AND (“public sector” OR “public administration”))

To qualify for the meta-analysis requirements, the retrieved articles must meet the following criteria. First, the research must be quantitative studies of the correlation between leadership styles and crisis management-related outcomes. Second, the study must incorporate the necessary effect size(s) (e.g., correlation coefficient (r) value, or t value (can be converted into r), and *p* value) (Lu et al., 2022). Third, the sample must be carried out within public administration or public organizations (e.g., civil servants, government agency employees, and public service workers). Studies conducted outside of PA contexts, such as general public surveys, student samples, or private sector samples, were excluded. Finally, the study must be a peer-reviewed journal article published in English to ensure analytical consistency (Luo et al., 2022). Conceptual papers, review articles, prior meta-analyses, dissertations, qualitative studies, and studies conducted outside public administration settings were excluded.

**Figure 2:**
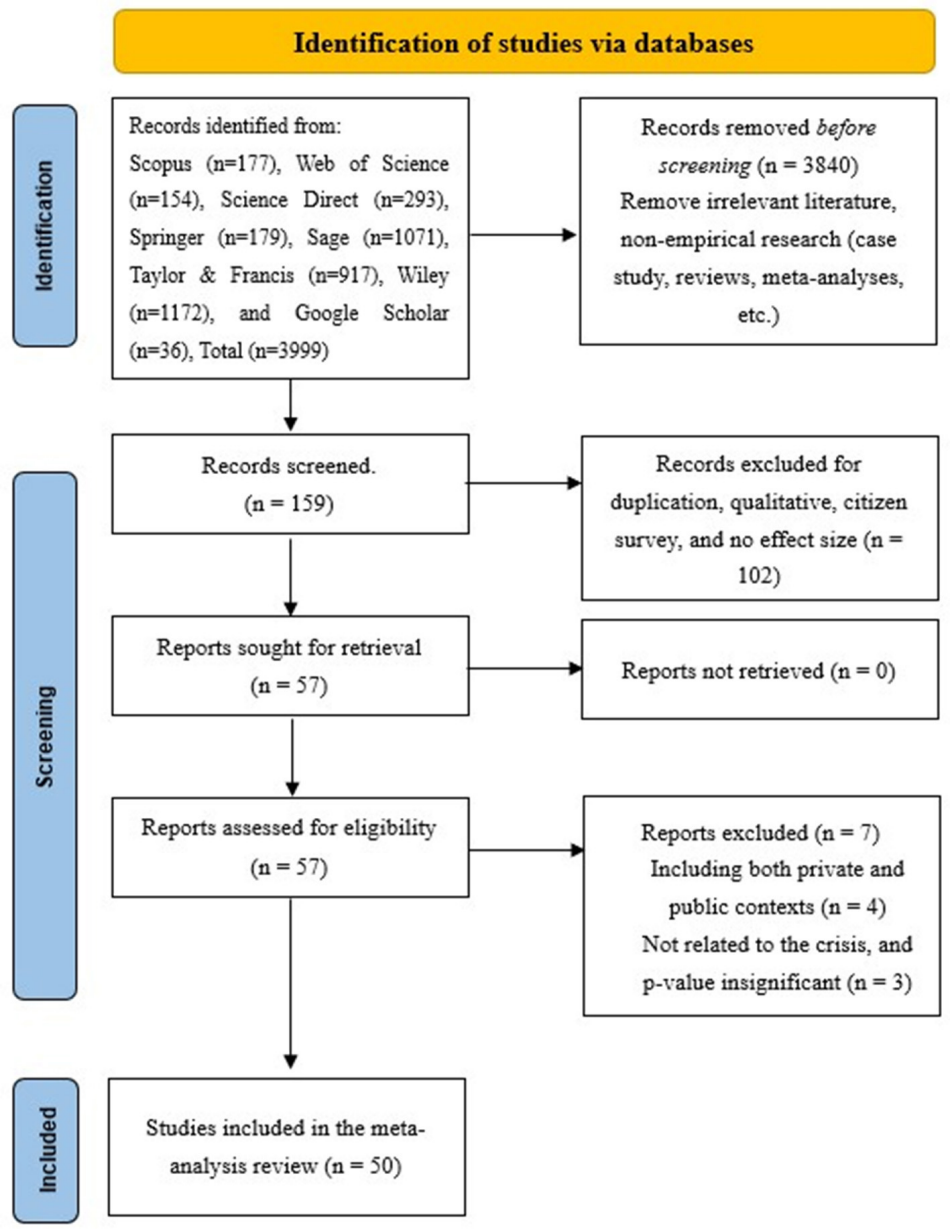
PRISMA flow diagram of the study selection.

The literature screening was performed in accordance with the PRISMA principles (Page et al., 2021). The initial database search yielded 3999 articles. Before screening, 3,840 records were removed because they were irrelevant to the topic or did not meet the basic study requirements, including non-empirical studies, case studies, reviews, and meta-analyses. The remaining 159 records were screened based on title and abstract, resulting in the exclusion of 102 records due to duplication, qualitative design, citizen-survey samples, or insufficient data for effect size calculation. Full texts were sought for 57 reports, all of which were successfully retrieved and assessed for eligibility. Of these, 7 reports were excluded because they combined private and public-sector contexts (*n* = 4) or were not sufficiently related to crisis management and did not provide usable evidence for the focal relationship (*n* = 3). After screening and assessment, 50 journal articles were included in the meta-analysis. These studies produced 72 effect sizes, 26 leadership style samples, and a total of 21815 participants (see Supplementary A). The PRISMA flow diagram is shown in Figure 2.

### 3.2 Coding procedure

Two researchers independently coded all eligible studies utilizing a standardized coding sheet. The extracted variables included: (i) bibliographical information (authors and publication year), (ii) sample and study characteristics (leadership styles, crisis management related outcomes, effect size, sample size, reliability, and relevant correlates), (iii) outcome construct classification, and (iv) moderator variables. The coding form was designed to ensure that study information was recorded in a structured and reproducible way. Transparent data collection procedures and explicit reporting of what data were sought are key elements of reproducible evidence synthesis (Dinnes et al., 2023). Specifically, crisis management-related outcomes were coded into conceptually distinct categories derived from the literature: overall crisis management-related outcomes, crisis-phase-related outcomes, sense-making, decision-making, meaning-making, and learning. Moderator variables included administrative subfields, administrative continent, crisis type, and participants' hierarchy level (see Supplementary B–F). To ensure coding reliability, the two coders worked independently and resolved discrepancies through discussion until consensus was reached. PRISMA 2020 recommends the specific reporting of the number of reviewers involved in data collection, their independence in the process, and the methods employed to resolve disagreements; thus, this revised description was incorporated for that purpose (Page et al., 2021). The conceptual definitions used to classify crisis management-related outcomes are shown in Table 3.

**Table 3:** Conceptual definitions of crisis management-related outcome constructs.

Construct	Description	Based on theory
Crisis management related outcomes	Broad indicators reflecting the extent to which public leaders and organizations manage crises effectively across crisis phases and core executive tasks (sense-making, decision-making, meaning-making, and continuous learning).	Boin et al., 2016; Pearson and Mitroff, 1993
Crisis phases	Outcomes related to crisis management phases, including three phases (pre-crisis, during crisis, and post-crisis), and five phases (signal detection, preparation and prevention, damage containment, recovery, and learning).	Pearson and Mitroff, 1993
Sense-making	Outcomes reflecting how leaders and organizations interpret crises, reduce ambiguity, and support resilience or psychological readiness.	Boin et al., 2016; Wolbers, 2021
Decision-making	Outcomes reflecting the quality or effectiveness of crisis decisions, including adaptability, agility, improvisation, coordination, organizing, and related decision processes.	Boin et al., 2016; Taheri, 2022
Meaning-making	Outcomes reflecting communication, credibility, trust, confidence, service recovery, and the construction of shared understanding during a crisis.	Boin et al., 2016; Jong, 2017
Learning	Outcomes reflecting the capacity to learn from crisis experience, improve competence, and support adaptation and future preparedness.	Boin et al., 2016; Eriksson and Hallberg, 2022

### 3.3 Risk of bias assessment

Two independent reviewers assessed the methodological quality and risk of bias for each study included in this review, using the Joanna Briggs Institute (JBI) critical appraisal checklist. The checklist includes eight components, and each item was rated as “yes”, “no”, or “unclear” (Alluhaybi et al., 2023; Moola et al., 2020). Disagreements regarding the ratings assigned by the reviewers were resolved through consensus. Based on the percentage of criteria met, studies were categorized as having low risk (≥70%), moderate risk (50–69%), or high risk (< 50%) (Gu and Cheng, 2026). The results revealed that the overall quality of the evidence base was high. Specifically, 44 studies (88%) were classified as having a low risk of bias, while 6 studies (12%) were classified as having a moderate risk of bias. No studies were excluded from the analysis based on the results of the methodological quality assessment. Detailed ratings for the methodological quality of each study are provided in Supplementary G.

### 3.4 Meta-analytical procedures

This research employed the comprehensive meta-analysis (CMA) version 3.0 (Lu et al., 2022; Lin et al., 2025; Nogueira et al., 2023). This study utilized a random effects model, which is appropriate for scenarios when respondents and measures differ, hence facilitating the generalization of results to similar studies (Fang and Zhang, 2018; Kasalak et al., 2022). Meta-analytic findings were evaluated using mean effect sizes, 95% confidence intervals, and *p*-values. The Pearson correlation coefficient (*r*) represents the common effect size metric. For studies reporting alternative statistics, effect sizes were converted to r where possible using the standard procedure.

Consistent with previous meta-analytic studies, this study preserved the multiple effect sizes when they represented conceptually distinct relationships within the same primary study (Backhaus and Vogel, 2022; Dunst et al., 2018; Borenstein et al., 2009; López-López et al., 2018; Hansen et al., 2021). This method preserved theoretical significance and minimized the risk of masking critical variations in leadership styles and crisis management outcomes. The primary analysis evaluated the overall effect of leadership styles on crisis management-related outcomes. Additional analyses were conducted for each conceptually distinct outcome category, namely crisis-phase-related outcomes, sense-making, decision-making, meaning-making, and learning. Although this strategy does not completely eradicate the statistical dependence among effect sizes from the same study, it mitigates the aggregation of conceptually overlapping associations and represents a pragmatic analytical approach considering the data structure (López-López et al., 2018; Cheung, 2019).

We also conducted moderator analyses within a random-effects framework to investigate potential sources of heterogeneity. The categorical moderators included administrative subfields, administrative continent, crisis types, and hierarchy level of participants. Administrative subfields were coded following Backhaus and Vogel (2022) as core administration, public education, public health, public services, and law enforcement/military. Administrative continent was coded as Asia, Europe, Africa, Australia, North America, South America, and Antarctica. Crisis types were coded into natural crises, civil conflicts, pandemics, and technological failures (Alzoubi and Jaaffar, 2020; Potočnik, 2025). Finally, participants' hierarchical levels were coded as senior, middle, or junior (Ansari and Naeem, 2010).

### 3.5 Publication bias assessment

Publication bias refers to “the tendency for studies with statistically significant results to be more likely to be published than studies reporting non-significant findings”, which can distort meta-analytic estimates (Rothstein et al., 2005). Such bias may arise not only from selective publication but also from selective reporting within studies and incomplete retrieval of relevant literature during the search process (Lin et al., 2025). To evaluate potential publication bias, the study utilized three complementary methods: the funnel plot visual method, Egger's regression coefficient test, and Begg's rank correlation test (Duval and Tweedie, 2000; Egger et al., 1997).

## 4 Results

### 4.1 Characteristics of included studies

**Figure 3:**
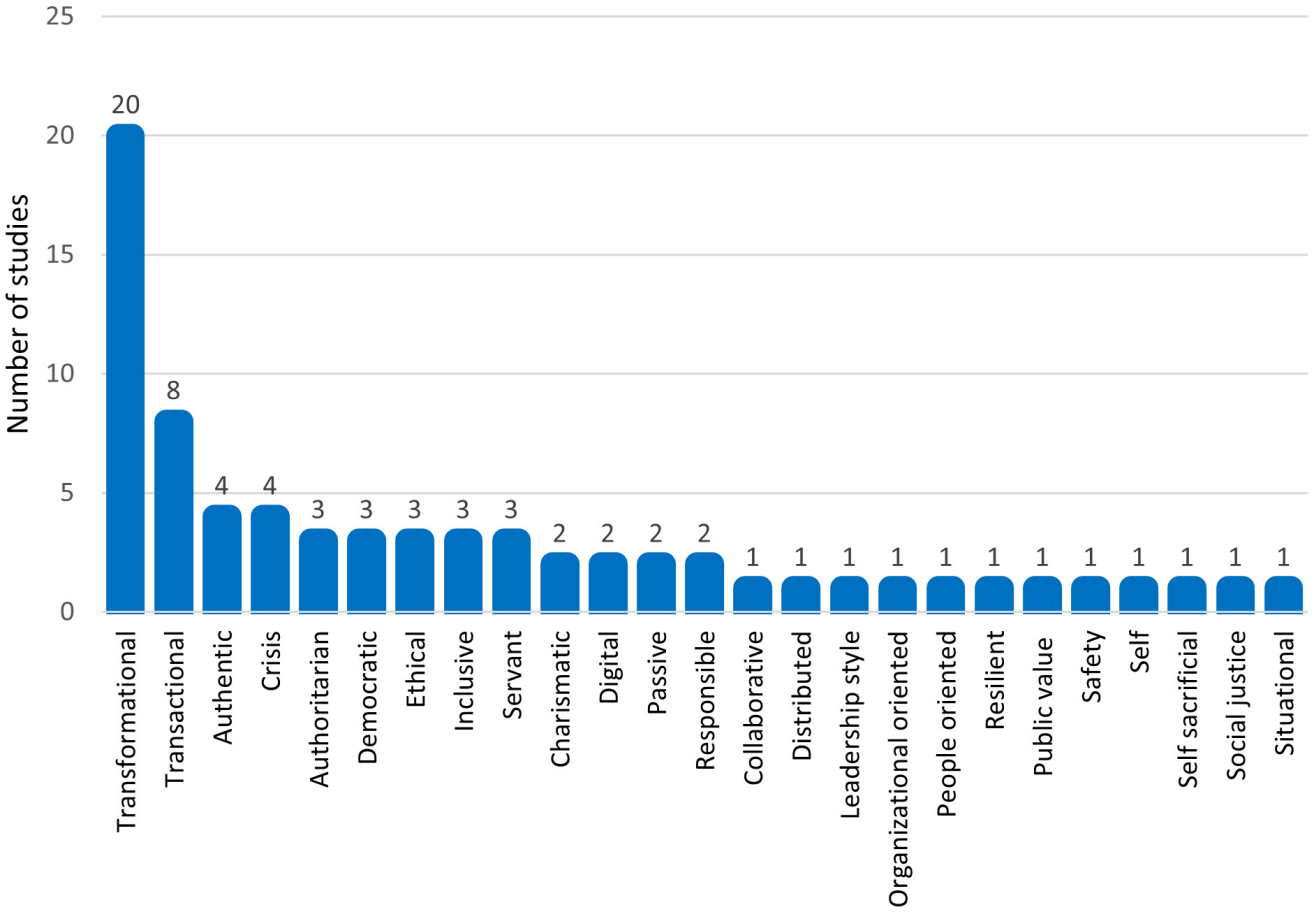
Distribution of leadership styles across the included studies.

This study first discovered the descriptive characteristics of the included research on leadership styles in public administration amid crisis management. The research discovered 72 effect sizes (*k* = 72) derived from 50 publications, representing 26 distinct leadership styles. Among these, transformational leadership was the most frequently investigated (*k* = 20). Subsequently, transactional leadership ranked second with a total of (*k* = 8). Afterward, other leadership styles, including crisis leadership, authentic leadership, digital leadership, inclusive leadership, and servant leadership, were represented by fewer than five effect sizes each. These findings suggest that transformational leadership remains the dominant focus of crisis-related leadership research in public administration. The distribution of leadership styles across the included studies is presented in Figure 3.

The contextual distribution of the included studies is presented in Table 4. Among the administrative continents, Asia comprised the predominant portion (*n* = 47, 65.3%). Europe constitutes a secondary focus area (*n* = 17, 24%), while research from Africa (*n* = 5, 7%), North America (*n* = 2, 2.8%), and Oceania (*n* = 1, 1.4%) is negligible. This pattern indicates a pronounced regional concentration in the current literature. The allocation of studies among administrative subfields reveals a significant concentration in public service settings, accounting for (*n* = 34, 47%). Public health is a notable secondary emphasis (*n* = 21, 29%), whilst public education (*n* = 9, 13%), core administration (*n* = 4, 6%), and law enforcement/military (*n* = 4, 6%) are relatively underexplored. The descriptive analysis of the crisis types in the nexus between leadership and crisis management shows that most academic studies have been done on pandemic-related research, including over half (*n* = 38, 53%) of the total 72 studies. Civil conflicts, which include man-made crises, also received substantial focus (*n* = 27, 38%), whereas natural crises (earthquakes, fires) were the subject of notably fewer studies (*n* = 7, 10%). This meta-analysis comprises three hierarchical levels. The study reveals that the majority of research focuses on junior-level employees, which accounts for the majority (*n* = 50, 69.4%). Middle-level (*n* = 14, 19.4%) and senior-level (*n* = 8, 11.1%) public officials receive substantially less attention when compared to junior-level officials. This imbalance suggests that current evidence is weighted more toward lower-level organizational perspectives than toward senior leadership roles.

**Table 4:** Descriptive characteristics of included studies.

Variables	Content	Frequency	Percentage
Administrative Continents	Africa	5	6.94
	Asia	47	65.28
	Europe	17	23.61
	North America	2	2.78
	Oceania	1	1.39
Administrative Subfields	Core Administration	4	5.56
	Public Education	9	12.50
	Public Health	21	29.17
	Law Enforcement	4	5.56
	Public Service	34	47.22
Types of Crises	Natural Crisis	7	9.72
	Civil Conflicts	27	37.50
	Pandemic	38	52.78
Hierarchy level	Junior level	50	69.44
	Middle level	14	19.44
	Senior level	8	11.11

### 4.2 Results of meta-analysis

Random-effects models were used to examine the relationship between leadership styles and six categories of crisis management-related outcomes. Table 5 presents the results.

**Table 5:** Results of the random effects model.

Model	k	Estimate	95% CI		z	p	Q	I^2^	Decision
			LB	UB					
H1	72	0.417	0.333	0.494	8.898	0.000	5110.047	98.611	Accepted
H2	14	0.426	0.291	0.544	5.740	0.000	348.789	96.273	Accepted
H3	19	0.313	0.102	0.498	2.865	0.004	2518.962	99.285	Accepted
H4	10	0.229	−0.020	0.451	1.804	0.071	519.701	98.268	Rejected
H5	13	0.614	0.400	0.765	4.803	0.000	896.231	98.661	Accepted
H6	16	0.456	0.334	0.563	6.658	0.000	511.441	97.067	Accepted

The first analysis examined the overall relationship between leadership styles and overall crisis management-related outcomes (H1). The mean effect size was positive and statistically significant (*r* = 0.417, 95% CI [0.333; 0.494], *z* = 8.898, *p* < 0.001), indicating a moderate positive association. However, substantial heterogeneity was observed (*Q* = 5110.047; *I*^2^ = 98.611%), suggesting that the variability in effect sizes cannot be attributed solely to sampling error. Specifically, the *I*^2^ value indicates that ~98.6% of the observed variance reflects real between-study differences rather than random fluctuation (Linden and Hönekopp, 2021). Such high heterogeneity is not unusual in organizational and leadership meta-analyses, where effect sizes may vary across institutional contexts, samples, crisis settings, and measurement strategies (Backhaus and Vogel, 2022; Choi and Kang, 2025). Therefore, the pooled estimate should be interpreted as an average effect across diverse contexts rather than as a universal effect size.

The second analysis examined the correlation between leadership styles and crisis-phase-related outcomes (H2). The effect size was again positive and statistically significant (*r* = 0.426, 95% CI: [0.291; 0.544], *z* = 5.740, *p* < 0.001), indicating a moderate positive association.

The third analysis assessed the impact of leadership styles on sense-making related outcomes (H3); the effect size is positive and statistically significant (*r* = 0.313, 95% CI: [0.102; 0.498], *z* = 2.865, *p* = 0.004), indicating a small to moderate positive association.

The fourth analysis examined the relationship between leadership styles and decision-making-related outcomes (H4). The pooled effect size was positive, and it was not statistically significant (*r* = 0.229, 95% CI: [−0.020; 0.451], *z* = 1.804, *p* = 0.071). Thus, the evidence did not support a reliable positive association between leadership styles and decision-making-related outcomes in the current meta-analysis.

The fifth analysis investigated the relationship between leadership styles and meaning-making related outcomes (H5). The mean effect size was positive and statistically significant (*r* = 0.614, 95% CI: [0.400; 0.765], *z* = 4.803, *p* < 0.001), indicating the strongest association among the outcome categories examined.

Finally, the sixth analysis examined the relationship between leadership styles and learning-related outcomes (H6). The mean effect size was positive and statistically significant (*r* = 0.456, 95% CI: [0.334; 0.563], *z* = 6.658, *p* < 0.000), indicating a moderate positive association. Therefore, we accepted hypotheses H1, H2, H3, H5, and H6, whereas H4 was rejected.

A methodological concern in the current meta-analysis is that some primary studies provided multiple relevant effect sizes. These were preserved when they expressed conceptually distinct relationships, such as different leadership styles within the same sample or multiple crisis management-related outcome categories reported in a single study (Lin et al., 2025; Lu et al., 2022; Backhaus and Vogel, 2022; Dunst et al., 2018; Nogueira et al., 2023). Preserving these effects upholds conceptually significant distinctions and prevents the loss of variance across leadership paradigms and outcome domains that could arise from aggregation. This strategy aligns with established meta-analytic practices, enhancing the dataset's comprehensiveness and analytical depth, while facilitating a more thorough examination of heterogeneity across constructs, contingent upon the proper acknowledgment and management of effect-size dependence (Borenstein et al., 2009; Lipsey and Wilson, 2000a,b; López-López et al., 2018; Moeyaert et al., 2016; Cheung, 2019).

Simultaneously, various effect sizes derived from the same sample may create statistical dependence. The analysis was performed in two phases to address this issue. An overall model was initially computed utilizing the complete array of suitable effect sizes related to crisis management outcomes. Subsequently, various random-effects models were estimated for conceptually different outcome domains, encompassing sense-making, decision-making, meaning-making, and learning. This approach does not entirely eliminate within-study dependence, but it mitigates the pooling of conceptually overlapping associations, hence serving as a pragmatic analytical strategy considering the available data and study design (Borenstein et al., 2009). Future studies can utilize higher-level methodologies, such as robust variance estimation or multilevel meta-analysis, contingent upon the structural suitability of the available data.

### 4.3 Results of moderator analyses

Moderator analyses were conducted to examine whether the relationship between leadership styles and overall crisis management-related outcomes varied across contextual conditions. To provide adequate statistical power, only moderator categories with a minimum of four correlation coefficients from primary research were selected in subgroup analysis (Lin et al., 2025). The results are reported in Table 6.

**Table 6:** Moderator analysis results.

Model	Moderator	Variables	*k*	Estimate	95% CI		*p*	*l* ^2^	Decision
					LB	UB			
H7	Administrative Subfield	Administration	4	0.460	0.148	0.689	0.005	97.356	Accepted
		Public Education	9	0.333	0.171	0.477	0.000	96.999	
		Public Health	21	0.453	0.287	0.592	0.000	98.792	
		Law Enforcement	4	0.123	−0.389	0.577	0.651	99.407	
		Public Service	34	0.441	0.324	0.546	0.000	98.296	
		Overall	72	0.409	0.332	0.481	0.000	98.611	
H8	Administrative continent	Africa	5	0.439	0.259	0.590	0.000	94.744	Accepted
		Asia	47	0.435	0.324	0.534	0.000	98.800	
		Europe	17	0.389	0.249	0.513	0.000	97.672	
		Overall	69	0.421	0.344	0.492	0.000	98.587	
H9	Types of Crises	Civil conflict	27	0.322	0.199	0.434	0.000	97.955	Accepted
		Natural crisis	7	0.383	0.257	0.497	0.000	93.436	
		Pandemic	38	0.484	0.353	0.596	0.000	98.965	
		Overall	72	0.388	0.316	0.456	0.000	98.611	
H10	Hierarchy Level	Junior level	50	0.475	0.378	0.561	0.000	98.612	Accepted
		Middle level	14	0.225	0.004	0.426	0.046	98.755	
		Senior level	8	0.353	0.147	0.529	0.001	96.915	
		Overall	72	0.412	0.331	0.488	0.000	98.611	

For administrative subfields (H7), the relationship between leadership styles and crisis management-related outcomes varied across public-sector domains. Significant positive associations were observed in core administration (*r* = 0.460, *p* = 0.005), public education (*r* = 0.333, *p* < 0.001), public health (*r* = 0.453, *p* < 0.001), and public service (*r* = 0.441, *p* < 0.001). By contrast, the relationship was weaker and non-significant in law enforcement (*r* = 0.123, *p* = 0.651). These findings suggest that the strength of the leadership-outcome relationship may differ by administrative subfield. Accordingly, H7 was supported.

For the administrative continent (H8), the leadership styles and outcomes correlation is weaker in Europe (*r* = 0.389, *p* < 0.001). Other continents, such as Africa (*r* = 0.439, *p* < 0.001) and Asia (*r* = 0.435, *p* < 0.001), have a significantly higher correlation. Although these patterns suggest some regional variation, they should be interpreted cautiously given the uneven geographic distribution of the evidence base. Therefore, the results provide support for H8.

For types of crises (H9), the leadership styles and outcomes relationship is significantly higher in a pandemic (*r* = 0.484, *p* < 0.001) than in a natural crisis (*r* = 0.383, *p* < 0.001) and in civil conflicts (*r* = 0.322, *p* < 0.001). These results suggest that the magnitude of the leadership-outcome relationship varies across crisis types. Therefore, H9 was supported.

Finally, for hierarchy level (H10), the relationship between leadership styles and outcomes was strongest among junior-level employees (*r* = 0.475, *p* < 0.001), weaker among senior-level participants (*r* = 0.353, *p* = 0.001), and weakest among middle-level participants (*r* = 0.225, *p* = 0.046). These findings suggest that leadership effects may be perceived or manifested differently across organizational levels. Thus, H10 was supported. Overall, the moderator analyses suggest that the relationship between leadership styles and crisis management-related outcomes is context-dependent rather than uniform across settings.

### 4.4 Results of publication bias

**Figure 4:**
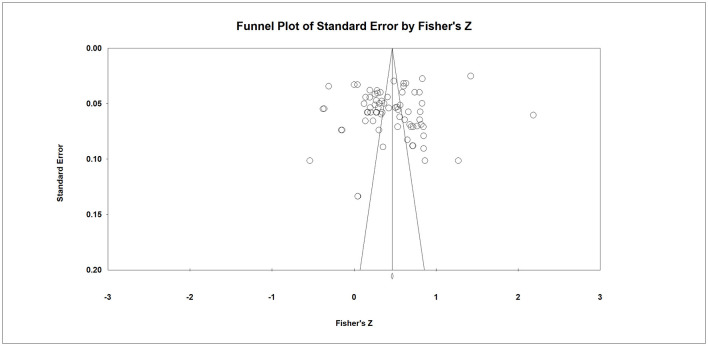
Funnel plot for the relationship between leadership styles and crisis management-related outcomes.

Publication bias was assessed using funnel plot inspection, Begg's rank correlation test, Egger's regression test, and Rosenthal's fail-safe N. The visual presentation of the funnel plot suggested some asymmetry, but statistical tests did not indicate significant small-study effects. Additionally, the results presented in Table 7, the *p*-values of Begg's rank correlation tests, and Egger's regression coefficient for leadership styles are not significant (*p* > 0.05). In addition, the fail-safe N was 7982, which substantially exceeded the conventional benchmark of 5k + 10 (i.e., >370 for *k* = 72). This indicates that a very large number of unpublished null-effect studies would be required to reduce the observed overall effect to non-significance. Consequently, these methods exhibit no indication of publication bias in the nexus between leadership styles and crisis management-related outcomes. Accordingly, further correction using the trim-and-fill method was not deemed necessary (Backhaus and Vogel, 2022). The funnel plot is presented in Figure 4.

**Table 7:** Publication bias assessment.

Variable	Begg's test		k	5k+10	Fail Safe N	Egger's intercept				
	**Z**	**p**				**Intercept**	**SE**	**t**	**df**	**p**
Leadership styles	1.05	0.29	72	370	7982.00	−2.93	3.00	0.97	70.00	0.33

## 5 Discussion

Although leadership has long been recognized as central to crisis management in public administration, the literature has remained fragmented, with studies focusing on different leadership styles, crisis contexts, and outcome constructs. The present meta-analysis was designed to provide a more integrated understanding of this relationship by synthesizing evidence across studies conducted in public-sector contexts. Drawing on 50 studies and 72 effect sizes, the findings indicate that leadership styles are, on average, positively associated with several crisis management-related outcomes in public administration. In particular, significant positive associations were found for overall crisis management-related outcomes, crisis-phase-related outcomes, sense-making, meaning-making, and learning. By contrast, the pooled relationship between leadership styles and decision-making-related outcomes was positive but not statistically significant. The moderator analyses further suggest that these relationships vary across administrative subfields, administrative continents, crisis types, and hierarchy levels. Overall, these findings significantly enhance the understanding of how leadership influences crisis management in public administration while also underscoring the contextual nature of leadership effectiveness.

Firstly, leadership in public administration amid crisis was expanding, and diverse studies. After the pandemic, the number of publications has risen compared to the past. In light of the effects of the COVID-19 pandemic, there is an increasing academic focus on the essential function of leadership in times of crisis (Collins et al., 2022). Among the leadership styles, transformational leadership got significant attention from various scholars, followed by transactional, authentic, and crisis leadership. Therefore, the study revealed that transformational leadership is a prevalent style in relation to crisis management under the PA context. This is understandable given that transformational leadership emphasizes inspiring, vision, inspirational motivation, individualized support, and intellectual stimulation, all of which are likely to be valued in conditions of uncertainty and disruption (Avolio and Bass, 1991; Bao et al., 2024). At the same time, the dominance of transformational leadership also reveals a limitation in the current literature. If the field focuses too heavily on one leadership paradigm, it may overlook alternative leadership styles that could be more effective in particular crisis conditions, institutional arrangements, or public-sector roles. Therefore, the findings highlight not only the prominence of transformational leadership but also the need for broader theoretical and empirical attention to a wider range of leadership styles.

Secondly, the meta-analysis finding revealed that leadership styles have a significant positive influence on most crisis management-related outcomes. The positive relationship with crisis-phase-related outcomes suggests that leadership supports the effective management of prevention, preparedness, response, and recovery processes across the crisis cycle (Gharib and Elnahas, 2021; Alene, 2022; Atilgan et al., 2025). This is consistent with the idea that crisis-oriented leadership behaviors, such as timely action, clear communication, coordination, and the effective use of resources, are especially valuable when public organizations operate under urgency and accountability pressures (Labib, 2024).

Leadership styles were also positively correlated with sense-making-related outcomes, suggesting that leaders facilitate common interpretation and situational clarity during times of crisis. This finding is consistent with prior work linking leadership to psychological safety, resilience, reduced distress, and improved interpretive capacity in turbulent contexts (Baran and Scott, 2010; Khoshlahn and Ardabili, 2016; Zhao et al., 2025; Rosing et al., 2022; Zhao et al., 2020). For instance, transformational and authentic leadership foster open communication and trust, motivating followers to engage actively with the situation and collaboratively cultivate a clear understanding of challenges during a crisis (Labib, 2024). In public administration, where crises often involve ambiguity, contested information, and the need for coordinated action across multiple actors, leadership may play a critical role in helping organizations understand events and establish a coherent response orientation.

Furthermore, leadership styles were positively associated with meaning-making related outcomes, suggesting that leadership matters not only for operational response but also for legitimacy, morale, and collective understanding. In crisis contexts, meaning-making involves communicating purpose, sustaining trust, preserving credibility, and helping employees and stakeholders interpret crisis events in ways that support coordinated action. The present findings are consistent with prior research linking leadership to organizational reputation, trust, quality of work life, workplace thriving, and wellbeing during crisis (Ebrahim et al., 2022; Huang and Zhou, 2023; Jamal and Bakar, 2015; Kriemadis and Despoteris, 2023). For instance, transformational leaders emphasize vision, values, and purpose, while transactional leaders concentrate on organizational structure and compliance with established protocols; both leadership styles are frequently effective during turbulent times that foster trust, provide clear purpose, perceive crises as opportunities for growth, and ensure organizational stability (Chiwisa, 2024). This pattern is especially relevant in public administration, where leadership is judged not only by technical efficiency but also by the extent to which it preserves public confidence and organizational cohesion during disruption.

Moreover, leadership styles substantially improve learning-related outcomes. Crisis management does not end with immediate response; it also requires reflection, adaptation, and the capacity to improve future performance. The present findings suggest that leadership may support such processes by encouraging knowledge sharing, team cohesion, productivity, innovation, workplace buoyancy, and adaptive capacity (Mao et al., 2024, 2025; Pham and Vu, 2023; Rafique et al., 2022; Yikilmaz et al., 2024; Zheng et al., 2015). This result aligns well with theoretical arguments that flexible, adaptive, and developmental forms of leadership are especially valuable under conditions of uncertainty and change (Ansell et al., 2022; Miao et al., 2025).

By contrast, the relationship between leadership styles and decision-making-related outcomes was positive but not statistically significant. This result does not necessarily imply that leadership is unimportant for crisis decision-making. Rather, it may reflect substantial variation in how decision-making was conceptualized and measured across the included studies, differences in crisis urgency and institutional constraints, or the possibility that leadership influences decision-making in more indirect or contingent ways than it influences other outcomes. In public administration, crisis decisions are often shaped not only by individual leadership behavior, but also by formal procedures, legal mandates, interagency coordination, and political oversight. These constraints may dilute or obscure the observable effect of leadership on decision-making-related outcomes in meta-analytic terms. Thus, the non-significant pooled estimate should be interpreted cautiously rather than as evidence that leadership does not matter for crisis decision-making.

Thirdly, the moderator analysis revealed that leadership effectiveness in crisis management appears to be context dependent. The moderator analyses suggest that the strength of the relationship between leadership styles and crisis management-related outcomes varies across administrative subfields, administrative continents, crisis types, and hierarchy levels. This pattern is consistent with contingency-based perspectives on leadership, which argue that no single leadership style is universally effective across all settings (Fiedler, 1964; Chiwisa, 2024). Specifically, the variation across administrative subfields reinforces the view that public-sector domains differ in their tasks, structures, professional norms, and service demands within the public sector, which may in turn shape how leadership is enacted and evaluated (Backhaus and Vogel, 2022). Moreover, cross-continental variation is consistent with the influence of broader cultural and administrative traditions on leadership expectations and crisis response practices (House et al., 2004; Backhaus and Vogel, 2022; Wang and Laufer, 2019). Similarly, Variation across crisis types further suggests that different crises impose different operational and interpretive demands, making some leadership approaches more compatible with particular crisis environments than others (Suharyanto and Lestari, 2020; Wu et al., 2021; Schaedler et al., 2021). Finally, differences across hierarchy levels imply that leadership may be experienced or assessed differently depending on organizational role and responsibility, which is consistent with prior research on hierarchical variation in leadership styles and functions (Ansari and Naeem, 2010). These findings suggest that average effect sizes are context-dependent rather than universal, supporting the application of a random-effects model and underscoring the necessity for future investigation into additional contextual and methodological sources of variation.

The significant heterogeneity noted across analyses constitutes a noteworthy finding. The relationship between leadership styles and outcomes connected to crisis management is not fixed; it fluctuates depending on context, sample size, crisis conditions, and measuring methods. This aligns with contingency theories of leadership, which assert that leadership effectiveness is contingent upon situational compatibility (Fiedler, 1964). Consequently, the averaged estimates need to be regarded as average effects across diverse research rather than as universal effects, and the application of a random-effects model was suitable since it accommodates variability in true effects among studies (Backhaus and Vogel, 2022; Bao et al., 2024; Lin et al., 2025). Although the moderator analyses explained some of this heterogeneity, residual variation persisted, suggesting that additional contextual and methodological factors demand investigation in future studies.

Overall, this meta-analysis provides a clearer and more integrated account of leadership in public administration during a crisis. The findings suggest that leadership styles are generally associated with stronger crisis management-related outcomes, but that these relationships are shaped by institutional and contextual conditions. The study therefore contributes to both theory and practice by clarifying that leadership matters in crisis management, while also showing that its effectiveness depends on where, when, and under what conditions it is exercised.

### 5.1 Implications for theoretical and managerial

This study provides several theoretical applications. Initially, it enhances the theory of leadership and crisis management by demonstrating that leadership should not be regarded as having a single, uniform influence on crisis management performance. Previous research often focused on either crisis phases or specific crisis-related outcomes (Maghdid et al., 2022; Tokakis et al., 2018; Aravidou et al., 2025; Jing and Yang, 2025). In the literature, crisis leadership has been conceptualized as a set of strategic tasks rather than a single outcome, especially in relation to sense-making, decision-making, meaning-making, and learning. This meta-analysis synthesizes evidence across many dimensions, illustrating that leadership styles varied in their relationships with distinct crisis management outcomes. The research advances a comprehensive theoretical understanding of crisis leadership as a multidimensional process, rather than only a one-dimensional predictor of crisis performance (Boin et al., 2016; Jong, 2017). Second, the findings reinforce a contingency-based perspective on leadership effectiveness in crises. Crisis management scholarship increasingly suggests that crisis management-related performance and leadership effectiveness cannot be interpreted apart from context, because uncertainty, urgency, institutional frameworks, and administrative environments shape the effectiveness of leadership styles. The moderator results show that the relationship between leadership styles and crisis management outcomes differs across administrative subfields, crisis types, administrative continents, and hierarchy levels. These findings therefore challenge one-size-fits-all assumptions and suggest that leadership effectiveness in crises is conditional on contextual and organizational factors (Van Wart, 2013; Deverell and Ganic, 2024).

Third, this study contributes to public administration theory by showing that crisis leadership in the public sector is shaped by the dual demands of governance capacity and legitimacy. Public leaders must coordinate actions, ensure service continuity, justify decisions, manage accountability, and uphold public trust amid increased scrutiny and institutional limitations. The study clarifies different relationships between leadership styles and crisis management outcomes in specific contexts, providing a more nuanced comprehension of crisis leadership within public organizations (Jong, 2017; Mao et al., 2025). Finally, the study highlights an important imbalance in the existing literature. The evidence base remains heavily concentrated on transformational leadership, while other leadership styles receive comparatively limited attention. This suggests that the current literature may provide a theoretically narrow account of crisis leadership in public administration. Future research should therefore expand the examination of less-studied leadership styles to develop a more balanced and comprehensive understanding of leadership effectiveness in crisis environments and public sector conditions (Riggio and Newstead, 2022).

This study also highlights several practical implications for public administration. First, the findings suggest that policymakers and public organizations should place greater emphasis on leadership development as part of crisis preparedness and response. In particular, leadership development initiatives should strengthen competencies related to communication, meaning-making, adaptability, and learning, as these dimensions are closely linked to effective crisis management. Second, the results suggest that public organizations should avoid relying on a single leadership model in all crisis contexts. Because leadership effectiveness varies across settings, leadership training and crisis management strategies should be tailored to the demands of particular administrative domains, crisis types, and hierarchy levels. A context-sensitive leadership approach is likely to be more effective than assuming that one leadership style will perform equally well across all public-sector crises. Finally, the findings indicate that strengthening leadership capacity can improve not only crisis response but also organizational resilience, employee wellbeing, and institutional adaptability. In this sense, leadership development should be considered part of long-term crisis governance rather than merely a reactive response mechanism.

### 5.2 Limitations and future research direction

While the study provides useful insights, several limitations should be acknowledged more explicitly. First, the present study selected journal articles published in the English language from eight databases to ensure the data quality. Future research should incorporate dissertations and other languages to broaden coverage and reduce publication or language bias. Second, this meta-analysis is based on quantitative studies to accomplish the study objectives. Some research papers employ a qualitative methodology in this domain. Future research should extend with a qualitative and mixed-method approach to achieve a comprehensive idea. Third, this meta-analysis was synthesized based on data availability across studies and included both beneficial and detrimental effects. In leadership studies, the majority of researchers have focused on the beneficial impact of leadership, while being limited in the detrimental impact (Backhaus and Vogel, 2022). Future studies should be conducted on beneficial and detrimental outcomes separately. Fourth, high heterogeneity was observed across the included studies, indicating substantial variation in contexts, measures, and research designs. Although moderator analyses were conducted, not all sources of heterogeneity could be fully explained. The findings should therefore be interpreted as average relationships across diverse contexts rather than universal estimates. Fifth, the evidence base was geographically concentrated, particularly in Asia and Europe, which may limit the global generalizability of the findings and introduce contextual bias. More evidence from underrepresented regions would help build a more balanced understanding of leadership in crisis management across public administration systems. Sixth, although the analyses were structured to reduce overlap across conceptually distinct relationships, some dependence among effect sizes derived from the same primary studies may remain. Future studies may apply advanced approaches, such as robust variance estimation or multilevel meta-analysis, to explicitly model within-study dependence among effect sizes. Finally, some crisis management-related outcomes may overlap conceptually, which could affect interpretation. Future studies should further refine these categories and apply more standardized coding frameworks.

## 6 Conclusion

This meta-analysis examined the relationship between leadership styles and crisis management-related outcomes in public administration, focusing on crisis phases, sense-making, decision-making, meaning-making, and learning. Based on 50 empirical studies, 72 effect sizes, 26 distinct leadership styles, and a total sample of 21,815 participants, the findings provide a stronger cumulative basis for inference than any single study alone. Overall, the findings reveal that leadership styles are positively associated with multiple dimensions of crisis management, particularly crisis phases, sense-making, meaning-making, and learning. Transformational leadership has emerged as the most extensively studied leadership style in the literature, highlighting its significance in public administration crisis studies. At the same time, the findings suggest that leadership effectiveness differs across crisis contexts. Rather, the strength of the relationship between leadership styles and crisis management-related outcomes varies across administrative subfields, administrative continents, crisis types, and hierarchy levels. These findings underscore the necessity for context-specific leadership development and crisis governance techniques in public administration. Leadership approaches should be aligned with the nature of the crisis and the institutional environment, and the organizational level at which leadership is exercised. Although this meta-analysis was derived from a quantitative approach, future research should extend with a qualitative and mixed-method approach to deepen the understanding of how leadership operates in crisis settings. Nevertheless, this meta-analysis reinforces the significance of leadership as a fundamental competency for public administration in crisis contexts and establishes a stronger foundation for future research and practice in crisis leadership.

## Data Availability

The original contributions presented in the study are included in the article/Supplementary material, further inquiries can be directed to the corresponding author.
