# Supplementary material for: Leadership in public administration amid crisis: a meta-analysis of leadership styles and crisis management related outcomes
Source: Front Psychol. 2026 May 18;17:1803778. doi: 10.3389/fpsyg.2026.1803778 (PMC13222819; doi:10.3389/fpsyg.2026.1803778)
Supplement: Supplementary file 1 [file Table_1.docx]

Supplementary Material

**Table of Contents**

Supplementary A. Studies included in meta-analysis2

Supplementary B. Coding scheme for crisis management outcomes6

Supplementary C. Coding scheme for administrative subfields8

Supplementary D. Coding scheme for administrative continents9

Supplementary E. Coding scheme for types of crises10

Supplementary F. Coding scheme for hierarchy levels11

Supplementary G. Risk of bias assessment12

Supplementary H. Data table14

**Supplementary A. Studies included in meta-analysis**

Ahmed, F., Zhao, F., & Faraz, N. A. (2020). How and When Does Inclusive Leadership Curb Psychological Distress During a Crisis? Evidence From the COVID-19 Outbreak. Frontiers in Psychology, 11, 1898. https://doi.org/10.3389/fpsyg.2020.01898

Ahmed, R. (2025). Resilient leadership, institutional adaptability, and crisis communication, and their role in emergency governance in Post-Pandemic public administration. Contemporary Public Administration Review, 2(2), 187–212. https://doi.org/10.26593/copar.v2i2.9376

AlAjmi, M. K. (2022). The impact of digital leadership on teachers’ technology integration during the COVID-19 pandemic in Kuwait. International Journal of Educational Research, 112, 101928. https://doi.org/10.1016/j.ijer.2022.101928

Alene, A. A. (2022). The influence of distributed leadership style on educational crisis management (the case of TPLF war) in government secondary schools of Bahir Dar City Administration, Ethiopia. Management in Education. https://doi.org/10.1177/08920206221139636

Alkharabsheh, A., Ahmad, Z. A., & Kharabsheh, A. (2014). Characteristics of crisis and decision-making styles: The mediating role of leadership styles. Procedia - Social and Behavioral Sciences, 129, 282–288. https://doi.org/10.1016/j.sbspro.2014.03.678

Al-Lawama, H. I., Darawsheh, S. R., Almahairah, M. S. Z., Al-Shaar, A. S., AlMahdawi, A. J., Shater, A., Gmach, I., Abdalla, H. O., & Alshurideh, M. T. (2023). The role of administrative leadership in crisis management in the Jordanian Ministry of Planning and International Cooperation. In Lecture notes on data engineering and communications technologies (pp. 447–458). https://doi.org/10.1007/978-3-031-27762-7_42

Aravidou, K., Triantari, S., & Zervas, I. (2025). Sustainable Leadership and Conflict Management: Insights from Greece’s Public Sector. Sustainability, 17(5), 2248. https://doi.org/10.3390/su17052248

Arboh, F., Dai, B., Quansah, P. E., Atingabilli, S., Drokow, E. K., & Addai‐Dansoh, S. (2024). Safety first, but how? Examining the impact of safety leadership in frontline healthcare workers’ safety performance during health crisis. Journal of Contingencies and Crisis Management, 32(3). https://doi.org/10.1111/1468-5973.12608

Atılgan, D., Öztürk, O., Temel, A. S., Akçakese, A., Tükel, Y., & Demirel, M. (2025). The role of authentic leadership in crisis situations: evidence from 2023 Kahramanmaraş earthquakes-affected and unaffected provinces in Türkiye. Environmental Hazards, 1–16. https://doi.org/10.1080/17477891.2025.2519313

Awad, N. H. A., & Ashour, H. M. A. (2022). Crisis, ethical leadership and moral courage: Ethical climate during COVID-19. Nursing Ethics, 29(6), 1441–1456. https://doi.org/10.1177/09697330221105636

Bataineh, O. T., Issa, H. M. B., & Al-Zoubi, Z. H. (2025). Situational leadership and its relationship to crisis management among Jordanian universities. International Journal of Evaluation and Research in Education (IJERE), 14(2), 777. https://doi.org/10.11591/ijere.v14i2.30405

Bellibaş, M. Ş., Polatcan, M., & Berkovich, I. (2025). Teachers’ self-efficacy, hope, resilience, and optimism: Linking social justice leadership, transformational leadership, and psychological capital. Educational Management Administration & Leadership. https://doi.org/10.1177/17411432251350794

Bellibaş, M. Ş., Polatcan, M., Berkovich, I., Boz, A., Bellibaş, M. Ş., Polatcan, M., Berkovich, I., & Boz, A. (2025). The role of responsible leadership in teacher resilience and wellbeing in Earthquake-Affected and Non-Affected regions. Educational Administration Quarterly. https://doi.org/10.1177/0013161x251396420

Ebrahim, Z. B., Hafidzuddin, S. A., Sauid, M. K., Mustakim, N. A., & Mokhtar, N. (2022). Leadership Style and Quality of Work Life among Nurses in Malaysia during the COVID-19 Pandemic Crisis. International Academic Symposium of Social Science 2022, 99. https://doi.org/10.3390/proceedings2022082099

Fahy, A., McCartney, S., Fu, N., & Roche, J. (2024). Investigating the indirect impact of transformational leadership on performance and work alienation: evidence from school principals navigating COVID-19. Leadership & Organization Development Journal, 45(5), 877–898. https://doi.org/10.1108/lodj-04-2022-0199

Gharib, A.E., & Elnahas, E. (2021). Evaluate The Role of Leadership in Crisis Management: A Field Study in Psychiatric Hospitals in Egypt. Global Business and Management Research: An International Journal. Vol. 13, No. 3 (2021). https://www.gbmrjournal.com/pdf/v13n3/V13N3-11.pdf

Habib, N., Naveed, S., Mumtaz, M., Sultana, R., & Akhtar, S. (2023). What type of leadership is more effective for managing change during force majeure? Achieving organizational effectiveness during the pandemic. RAUSP Management Journal, 58(4), 318–340. https://doi.org/10.1108/rausp-01-2023-0007

Harb, B., Hachem, B., & Hamdan, H. (2020). Public servants’ perception of leadership style and its impact on organizational commitment. Problems and Perspectives in Management, 18(4), 319–333. https://doi.org/10.21511/ppm.18(4).2020.26

Hasan, A., & Rjoub, H. (2017). The role of effective leadership styles in crisis management: A study of ERBIL, IRAQ. International Journal of Economics, Commerce and Management. https://ijecm.co.uk/wp-content/uploads/2017/04/548a.pdf

Huang, D., & Zhou, H. (2023). Self-sacrificial leadership, thriving at work, workplace well-being, and work–family conflict during the COVID-19 crisis: The moderating role of self-leadership. BRQ Business Research Quarterly, 27(1), 10–25. https://doi.org/10.1177/23409444231203744

Jamal, J., & Bakar, H. A. (2015). The mediating role of charismatic leadership communication in a crisis: A Malaysian example. International Journal of Business Communication, 54(4), 369–393. https://doi.org/10.1177/2329488415572782

Juma, A. a. Z. O., Batool, M., Ali, S., & Perumal, P. A. (2022). The impact of transformational leadership on risk management and Dubai police performance. Policing a Journal of Policy and Practice, 17. https://doi.org/10.1093/police/paac060

Juma, A. a. Z. O., Peruma, P. A., & Mansoor, N. (2022). Model of Transformational Leadership, Risk management factors influencing on law performance. International Journal of Sustainable Construction Engineering Technology, 13(4). https://doi.org/10.30880/ijscet.2022.13.04.026

Kapucu, N., & Demirhan, C. (2017). Managing collaboration in public security networks in the fight against terrorism and organized crime. International Review of Administrative Sciences, 85(1), 154–172. https://doi.org/10.1177/0020852316681859

Khoshlahn, M., & Ardabili, F. S. (2016). The role of organizational agility and transformational leadership in service Recovery prediction. Procedia - Social and Behavioral Sciences, 230, 142–149. https://doi.org/10.1016/j.sbspro.2016.09.018

Kjeldsen, A. M., Stennicke, M. G., Gregersen, D. S., Petersen, C. L., Bager, A. V., Jønsson, T. F., & Andersen, L. B. (2023). Crisis intensity, leadership behavior, and employee outcomes in public organizations. International Journal of Public Administration, 47(14), 951–970. https://doi.org/10.1080/01900692.2023.2213856

Kriemadis, A., & Despoteris, G. (2023). Leadership and effectiveness: the case of public sector during the COVID-19 pandemic crisis period. International Journal of Applied Systemic Studies, 1(1). https://doi.org/10.1504/ijass.2023.10057945

Ly, B., & Ly, R. (2024). Technostress in times of change: unveiling the impact of leadership styles in Cambodia’s public organizations in the wake of COVID-19. Cogent Business & Management, 11(1). https://doi.org/10.1080/23311975.2024.2331645

Ma, Y., Faraz, N. A., Ahmed, F., Iqbal, M. K., Saeed, U., Mughal, M. F., & Raza, A. (2021). Curbing nurses’ burnout during COVID‐19: The roles of servant leadership and psychological safety. Journal of Nursing Management, 29(8), 2383–2391. https://doi.org/10.1111/jonm.13414

Mao, Q., Fan, C., & Wang, X. (2024). The impact of crisis leadership on the performance in public sector: The mediation role of trust and knowledge sharing. Journal of Contingencies and Crisis Management, 32(3). https://doi.org/10.1111/1468-5973.12610

Mao, Q., Zhang, Y., & Fan, C. (2025). How can crisis leadership encourage civil servant performance? the mediating role of knowledge sharing, trust and public service motivation. Humanities and Social Sciences Communications, 12(1). https://doi.org/10.1057/s41599-025-04519-2

Maruhom, A. P., Minoza, J. T., & Zosa, L. T. (2024). Transformational Leadership and Crisis Management Practices of Cebu Technological University-Tuburan. International Journal of Multidisciplinary Applied Business and Education Research, 5(3), 863–877. https://doi.org/10.11594/ijmaber.05.03.11

Mohammad, H. F., Hashish, E. a. A., & Elliethey, N. S. (2023). The relationship between authentic leadership and nurses’ resilience: a mediating role of Self-Efficacy. SAGE Open Nursing, 9, 23779608231214213. https://doi.org/10.1177/23779608231214213

Naser, M., Alharthi, A., & Khalifa, G.S. (2019). Business Continuity Management and Crisis Leadership: An Approach to Re-Engineer Crisis Performance within Abu Dhabi Governmental Entities. International Journal on Emerging Technologies 10(1a): 32-40(2019). https://www.researchtrend.net/ijet/pdf/Business%20Continuity%20Management%20and%20Crisis%20Leadership.pdf

Ndone, J. (2025). Public relations in the age of AI: the moderating effects of authentic leadership and supervisors’ digital literacy on AI-induced uncertainty among Kenyan public relations practitioners. Corporate Communications an International Journal, 1–22. https://doi.org/10.1108/ccij-03-2025-0057

Obuobisa-Darko, T., Asiedu, E. A., Ohemeng, F., & Parku, K. (2023). Managing employee engagement in crisis situations in developing countries: The case for Responsible Leadership in the Public sector in Ghana. Public Organization Review, 24(3), 803–821. https://doi.org/10.1007/s11115-023-00733-1

Pham, H. Q., & Vu, P. K. (2023). Does public value commitment leadership and corporate social responsibility fuel accountants’ productivity during the COVID-19 pandemic and new normal: A case study on the public sector in Vietnam. Public Organization Review, 23(2), 575–603. https://doi.org/10.1007/s11115-023-00714-4

Rafique, M. A., Hou, Y., Chudhery, M. a. Z., Waheed, M., Zia, T., & Chan, F. (2022). Investigating the impact of pandemic job stress and transformational leadership on innovative work behavior: The mediating and moderating role of knowledge sharing. Journal of Innovation & Knowledge, 7(3), 100214. https://doi.org/10.1016/j.jik.2022.100214

Rosing, F., Boer, D., & Buengeler, C. (2022). When timing is key: How autocratic and democratic leadership relate to follower trust in emergency contexts. Frontiers in Psychology, 13, 904605. https://doi.org/10.3389/fpsyg.2022.904605

Salehi, N., Brunetto, Y., & Dick, T. (2023). The role of authentic leadership on healthcare Street‐Level Bureaucrats’ well‐being during the pandemic. Australian Journal of Public Administration, 82(2), 271–289. https://doi.org/10.1111/1467-8500.12584

Taie, E. S., & Zoromba, M. A. (2022). The COVID-19 pandemic highlights the importance of inclusive leadership in Egyptian hospitals to improve nurses’ psychological distress. Psych, 4(3), 537–548. https://doi.org/10.3390/psych4030041

Williams, E., Woods, J. M., Hertelendy, A., & Kloepfer, K. (2019). Supervisory influence. Journal of Organizational Change Management, 32(3), 320–339. https://doi.org/10.1108/jocm-10-2017-0373

Yikilmaz, I., Surucu, L., Maslakci, A., Dalmis, A. B., & Ergun, M. (2024). Workplace buoyancy and servant leadership as catalysts for sustainable disaster management: Mitigating emotional exhaustion in disaster response teams. Sustainability, 16(7), 2695. https://doi.org/10.3390/su16072695

Zada, M., Zada, S., Khan, J., Saeed, I., Zhang, Y. J., Vega-Muñoz, A., & Salazar-Sepúlveda, G. (2022). Does servant leadership control psychological distress in crisis? Moderation and mediation mechanism. Psychology Research and Behavior Management, Volume 15, 607–622. https://doi.org/10.2147/prbm.s354093

Zadok, A., Benoliel, P., & Schechter, C. (2024). School middle leaders’ transformational leadership and organizational resilience: The moderating role of academic emphasis. European Journal of Education, 59(3). https://doi.org/10.1111/ejed.12657

Zeer, I.A., Salahat, M., Ajouz, M., Siaj, R., Alsabatin, H., Alramahi, N.M., Tunsi, W. (2025). Strategic Planning and Leadership Styles as the Key Mediators in Change Management and Crisis Management in the Higher Education Sector. International Journal of Operations and Quantitative Management. Volume 31, Number 1, March 2025, pp. 141-161. https://submissions.ijoqm.org/index.php/ijoqm/article/view/435

Zhang, M., Chen, H., Wang, N., Li, Y., & Liu, Y. (2022). Does transformational leadership and psychological empowerment improve nurses’ innovative behaviour during COVID‐19 outbreak? A cross‐sectional study. Journal of Nursing Management, 30(8), 4116–4125. https://doi.org/10.1111/jonm.13877

Zhao, F., Ahmed, F., & Faraz, N. A. (2020). Caring for the caregiver during COVID-19 outbreak: Does inclusive leadership improve psychological safety and curb psychological distress? A cross-sectional study. International Journal of Nursing Studies, 110, 103725. https://doi.org/10.1016/j.ijnurstu.2020.103725

Zhao, G., Zhao, F., Hui, X., Wu, Y., & Zhao, X. (2025). How digital leadership impacts community resilience: a moderated mediation model. Frontiers in Public Health, 13, 1524985. https://doi.org/10.3389/fpubh.2025.1524985

Zheng, D., Witt, L., Waite, E., David, E. M., Van Driel, M., McDonald, D. P., Callison, K. R., & Crepeau, L. J. (2015). Effects of ethical leadership on emotional exhaustion in high moral intensity situations. The Leadership Quarterly, 26(5), 732–748. https://doi.org/10.1016/j.leaqua.2015.01.006

**Supplementary B. Coding scheme for outcomes**

| **No** | **Outcome/Correlate** | **Outcome type** |
| --- | --- | --- |
| 1 | Administrative and instructional use | Sense making |
| 2 | Agility | Sense making |
| 3 | Avoidance | Decision making |
| 4 | Benevolence | Meaning making |
| 5 | Burnout | Sense making |
| 6 | Business continuity management | Decision making |
| 7 | Communication use | Sense making |
| 8 | Community resilience | Sense making |
| 9 | Comprehensive decision-making | Decision making |
| 10 | Compromise | Decision making |
| 11 | Confrontation | Decision making |
| 13 | Corporate social responsibility | Meaning making |
| 14 | Crisis Intensity-Disease | Sense making |
| 15 | Crisis Intensity-Lockdown | Sense making |
| 16 | Crisis Intensity-Restriction | Sense making |
| 17 | Crisis leader potential | Sense making |
| 18 | Crisis management | Crisis phase |
| 19 | Crisis responsibility | Meaning making |
| 20 | Effectiveness | Learning |
| 21 | Effectiveness of crisis leadership | Learning |
| 22 | Emergency governance effectiveness | Decision making |
| 23 | Emotional exhaustion | Sense making |
| 24 | Employee well-being | Meaning making |
| 25 | Extra Effort | Sense making |
| 26 | Innovative behaviour | Learning |
| 27 | Innovative work behaviour | Learning |
| 28 | Job performance | Learning |
| 29 | Knowledge sharing | Learning |
| 30 | Law enforcement performance | Decision making |
| 31 | Moral courage | Decision making |
| 32 | Organising | Decision making |
| 33 | Organizational commitment | Meaning making |
| 34 | Organizational effectiveness | Learning |
| 35 | Organizational reputation | Meaning making |
| 36 | Organizational resilience | Sense making |
| 37 | Performance | Learning |
| 38 | Productivity | Learning |
| 39 | Psychological capital | Meaning making |
| 40 | Psychological distress | Sense making |
| 41 | Psychological safety | Sense making |
| 42 | Public service motivation | Meaning making |
| 43 | Quality of work life | Meaning making |
| 44 | Resilience | Learning |
| 45 | Restrictive decision-making | Decision making |
| 46 | Risk management | Sense making |
| 47 | Safety consciousness | Sense making |
| 48 | Safety participation | Meaning making |
| 49 | Satisfaction | Meaning making |
| 50 | Self efficacy | Meaning making |
| 51 | Service recovery | Learning |
| 53 | Team cohesion | Learning |
| 54 | Technology integration | Learning |
| 55 | Technostress | Sense making |
| 56 | Thriving at work | Meaning making |
| 57 | Trust | Sense making |
| 58 | Trust in the leader | Sense making |
| 59 | Well being | Meaning making |
| 60 | Work alienation | Sense making |
| 61 | Work engagement | Meaning making |
| 62 | Work-family conflict | Meaning making |
| 63 | Workplace buoyancy | Learning |
| 64 | Workplace well-being | Meaning making |

**Supplementary C. Coding scheme for administrative subfields**

| **No** | **Context** | **Administrative subfields** |
| --- | --- | --- |
| 1 | Bank | Public Service Sector |
| 2 | Civil Defense | Law Enforcement |
| 3 | Department of Finance | Public Service Sector |
| 4 | Department of Public Works | Public Service Sector |
| 5 | Disaster Management Unit | Public Service Sector |
| 6 | Emergency Governance Unit | Public Service Sector |
| 7 | Fire Fighting Department | Public Service Sector |
| 8 | Government entities | Public Service Sector |
| 9 | Government Organizations | Public Service Sector |
| 10 | Lowest administrative level | Core Administration |
| 11 | Military | Law Enforcement |
| 12 | Ministry of Interior | Core Administration |
| 13 | Ministry of Planning | Public Service Sector |
| 14 | Ministry of planning and international cooperation | Public Service Sector |
| 15 | Police | Law Enforcement |
| 16 | Public Administration Department | Core Administration |
| 17 | Public Education | Public Education |
| 18 | Public Health | Public Health |
| 19 | Public Organization | Public Service Sector |
| 20 | Public Sector | Public Service Sector |

**Supplementary D. Coding scheme for administrative continents**

| **No** | **Country** | **Continent** |
| --- | --- | --- |
| 1 | Australia | Ocenia |
| 2 | Cambodia | Asia |
| 3 | China | Asia |
| 4 | Denmark | Europe |
| 5 | Dubai | Asia |
| 6 | Egypt | Asia |
| 7 | Ethiopia | Africa |
| 8 | Germany | Europe |
| 9 | Ghana | Africa |
| 10 | Greece | Europe |
| 11 | Iran | Asia |
| 12 | Iraq | Asia |
| 13 | Ireland | Europe |
| 14 | Israel | Asia |
| 15 | Jordan | Asia |
| 16 | Kenya | Africa |
| 17 | Kuwait | Asia |
| 18 | Lebanon | Asia |
| 19 | Malaysia | Asia |
| 20 | Pakistan | Asia |
| 21 | Palestine | Asia |
| 22 | Philippines | Asia |
| 23 | Turkey | Europe |
| 24 | UAE | Asia |
| 25 | USA | North America |
| 26 | Vietnam | Asia |

**Supplementary E. Coding scheme for types of crises**

| **No** | **Crises** | **Major type** |
| --- | --- | --- |
| 1 | Crime | Civil conflict |
| 2 | Earthquake | Natural crisis |
| 3 | Fire | Natural crisis |
| 4 | General crisis | Civil conflict |
| 5 | Military operation | Civil conflict |
| 6 | Organizational crisis | Civil conflict |
| 7 | Pandemic | Pandemic |
| 8 | Political and Economic | Civil conflict |

**Supplementary F. Coding scheme for hierarchy levels**

| **No** | **Participant position** | **Hierarchy level** |
| --- | --- | --- |
| 1 | Academic staffs | Junior |
| 2 | Accountant | Junior |
| 3 | Administrative personnel | Junior |
| 4 | Civil Defense Officers | Middle |
| 5 | Civil Servants | Junior |
| 6 | Disaster Response Workers | Junior |
| 7 | Employees | Junior |
| 8 | Employees and Supervisors | Middle |
| 9 | Firefighters | Junior |
| 10 | Frontline health workers | Junior |
| 11 | Head of department, manager, senior executive | Senior |
| 12 | Health care staff | Junior |
| 13 | Managers | Middle |
| 14 | Middle Personnel | Middle |
| 15 | Middle Public Managers | Middle |
| 16 | Nurses | Junior |
| 17 | Nurses, Senior Nurses | Middle |
| 18 | Police officers | Middle |
| 19 | Principals | Senior |
| 20 | Principals and Teachers | Middle |
| 21 | Professor and Associate Professor | Senior |
| 22 | Public Managers | Middle |
| 23 | Senior Administrative Authorities | Senior |
| 24 | Senior Nurses | Senior |
| 25 | Teachers | Junior |
| 26 | Teachers and middle leaders | Middle |
| 27 | Urban Communities Workers | Junior |

**Supplementary G. Risk of bias assessment**

Please see the uploaded file metadata.xlsx*.*

| **No** | **JBI Questions** |
| --- | --- |
| 1 | Were the criteria for inclusion in the sample clearly defined? |
| 2 | Were the study subjects and the setting described in detail? |
| 3 | Was the exposure measured in a valid and reliable way? |
| 4 | Are objective, standard criteria used for measurement of the condition? |
| 5 | Were confounding factors identified? |
| 6 | Were strategies to deal with confounding factors stated? |
| 7 | Were the outcomes measured in a valid and reliable way? |
| 8 | Was appropriate statistical analysis used? |

| **ID** | **Author and Year** | **Number of "yes" answers** | **Total number of questions** | **Quality Score (%)** | **Risk of bias** |
| --- | --- | --- | --- | --- | --- |
| 1 | Gharib & Elnahas, 2021 | 5 | 8 | 63% | Moderate risk |
| 2 | Hasan & Rjoub, 2017 | 5 | 8 | 63% | Moderate risk |
| 3 | Zeer et al., 2025 | 5 | 8 | 63% | Moderate risk |
| 4 | Alene, 2022 | 6 | 8 | 75% | Low risk |
| 5 | Al-Lawama et al., 2023 | 5 | 8 | 63% | Moderate risk |
| 6 | Ly & Ly, 2024 | 8 | 8 | 100% | Low risk |
| 7 | Zada et al., 2022 | 7 | 8 | 88% | Low risk |
| 8 | Arboh et al., 2024 | 7 | 8 | 88% | Low risk |
| 9 | Mao et al., 2024 | 7 | 8 | 88% | Low risk |
| 10 | Fahy et al., 2024 | 7 | 8 | 88% | Low risk |
| 11 | Zhao et al., 2020 | 6 | 8 | 75% | Low risk |
| 12 | Habib et al., 2023 | 6 | 8 | 75% | Low risk |
| 13 | Yikilmaz et al., 2024 | 6 | 8 | 75% | Low risk |
| 14 | G. Zhao et al., 2025 | 7 | 8 | 88% | Low risk |
| 15 | AlAjmi, 2022 | 6 | 8 | 75% | Low risk |
| 16 | Bellibaş et al., 2025 | 7 | 8 | 88% | Low risk |
| 17 | Kapucu & Ustun, 2017 | 6 | 8 | 75% | Low risk |
| 18 | Khoshlahn & Ardabili, 2016 | 6 | 8 | 75% | Low risk |
| 19 | Mao et al., 2025 | 7 | 8 | 88% | Low risk |
| 20 | Aravidou et al., 2025 | 6 | 8 | 75% | Low risk |
| 21 | Ahmed, 2025 | 6 | 8 | 75% | Low risk |
| 22 | Harb et al., 2020 | 6 | 8 | 75% | Low risk |
| 23 | Jamal & Bakar, 2015 | 7 | 8 | 88% | Low risk |
| 24 | Rosing et al., 2022 | 7 | 8 | 88% | Low risk |
| 25 | Maruhom et al., 2024 | 6 | 8 | 75% | Low risk |
| 26 | Bataineh et al., 2025 | 5 | 8 | 63% | Moderate risk |
| 27 | Kriemadis & Despoteris, 2023 | 6 | 8 | 75% | Low risk |
| 28 | Alkharabsheh et al., 2014 | 6 | 8 | 75% | Low risk |
| 29 | Juma et al., 2022 | 5 | 8 | 63% | Moderate risk |
| 30 | Alharthi & Khalifa, 2019 | 6 | 8 | 75% | Low risk |
| 31 | Williams et al., 2019 | 6 | 8 | 75% | Low risk |
| 32 | Rafique et al., 2022 | 6 | 8 | 75% | Low risk |
| 33 | Obuobisa-Darko et al., 2023 | 6 | 8 | 75% | Low risk |
| 34 | Pham & Vu, 2023 | 6 | 8 | 75% | Low risk |
| 35 | Kjeldsen et al., 2023 | 7 | 8 | 88% | Low risk |
| 36 | Juma, Peruma, et al., 2022 | 6 | 8 | 75% | Low risk |
| 37 | Huang & Zhou, 2023 | 7 | 8 | 88% | Low risk |
| 38 | Ma et al., 2021 | 7 | 8 | 88% | Low risk |
| 39 | Atılgan et al., 2025 | 7 | 8 | 88% | Low risk |
| 40 | F. Ahmed et al., 2020 | 7 | 8 | 88% | Low risk |
| 41 | Ebrahim et al., 2022 | 6 | 8 | 75% | Low risk |
| 42 | Zhang et al., 2022 | 7 | 8 | 88% | Low risk |
| 43 | Zadok et al., 2024 | 7 | 8 | 88% | Low risk |
| 44 | Zheng et al., 2015 | 6 | 8 | 75% | Low risk |
| 45 | Awad & Ashour, 2022 | 7 | 8 | 88% | Low risk |
| 46 | Bellibaş et al., 2025 | 6 | 8 | 75% | Low risk |
| 47 | Mohammad et al., 2023 | 6 | 8 | 75% | Low risk |
| 48 | Taie & Zoromba, 2022 | 6 | 8 | 75% | Low risk |
| 49 | Salehi et al., 2023 | 7 | 8 | 88% | Low risk |
| 50 | Ndone, 2025 | 7 | 8 | 88% | Low risk |

**Supplementary H. Data table**

*Please see the uploaded file supplementary2.xlsx.*
